# Physical, Chemical and Biochemical Modifications of Protein-Based Films and Coatings: An Extensive Review

**DOI:** 10.3390/ijms17091376

**Published:** 2016-08-23

**Authors:** Joël Zink, Tom Wyrobnik, Tobias Prinz, Markus Schmid

**Affiliations:** 1 Fraunhofer Institute for Process Engineering and Packaging IVV, Giggenhauser Strasse 35, Freising 85354, Germany; 2 Chair of Food Packaging Technology, Technische Universität München, Weihenstephaner Steig 22, Freising 85354, Germany

**Keywords:** protein-based films, protein-based coatings, whey protein, soy protein, wheat gluten protein, mechanical properties, barrier properties, physical modification, chemical modification, biochemical modification

## Abstract

Protein-based films and coatings are an interesting alternative to traditional petroleum-based materials. However, their mechanical and barrier properties need to be enhanced in order to match those of the latter. Physical, chemical, and biochemical methods can be used for this purpose. The aim of this article is to provide an overview of the effects of various treatments on whey, soy, and wheat gluten protein-based films and coatings. These three protein sources have been chosen since they are among the most abundantly used and are well described in the literature. Similar behavior might be expected for other protein sources. Most of the modifications are still not fully understood at a fundamental level, but all the methods discussed change the properties of the proteins and resulting products. Mastering these modifications is an important step towards the industrial implementation of protein-based films.

## 1. Introduction

The importance of food packaging materials has increased enormously over the last 70 years during the change from a society based on rural self-sufficiency to the highly industrialized food industry of today. The focus has constantly been the protection of foods from mechanical damage, water vapor, and oxygen and the improvement of the shelf life of the packaged goods [1,2]. Accordingly, interest in protein-based films and coatings has increased considerably over recent years due to their advantages over conventional petroleum-based materials and over other biological materials such as polysaccharides and lipids [3]. Protein-based materials are usually biodegradable and are extracted from renewable sources. Furthermore, proteins are generally superior to polysaccharides in their ability to form films with high mechanical and barrier properties and they provide higher nutritional value [4,5]. Moreover, they can be used for controlled release of additives and bioactive compounds [6,7,8,9]. Thus, many studies have been conducted in order to produce films and coatings from various protein sources such as whey [10,11,12,13], soy [14,15], wheat gluten (WG) [16,17], pea [18], and amaranth [19]. Whey, along with soy and wheat gluten proteins (WG), is one of the most interesting materials as it is one of the world’s largest and essentially unexploited protein sources [5,20]. Soy and wheat gluten proteins are also abundantly available [21,22,23,24].

Protein-based films and coatings have good mechanical, optical, and oxygen barrier properties but also have high sensitivity to moisture and poor water vapor barrier properties due to their hydrophilic nature [25,26]. The required characteristics of a packaging material vary with its intended use and thus have to be customized. Many studies have therefore been conducted to optimize the functional properties of protein-based films and coatings using a variety of methods [27,28,29].

The aim of this review is to provide an overview of the effects and mechanisms of the physical, chemical, and biochemical treatment of protein-based films and coatings. Special attention is placed on the mechanical and barrier properties and the emphasis is put on whey, soy, and wheat gluten proteins which are among the most widely studied.

## 2. Protein Films and Coatings

### 2.1. Definition and Characteristics of Proteins

Proteins are organic macromolecules composed of α-amino acids which are linked by peptide bonds to form the primary structure. They differ from peptides by their larger size of approximately 100 amino acids. The polypeptide backbone is essential for the folding of the protein which is designated as the secondary structure. Stabilized by intramolecular interactions, α-helices and β-sheets are established as well as loops and bends. The tertiary structure is the global configuration of proteins, resulting from intermolecular interactions of the protein side chains. Moreover, certain proteins develop a quaternary structure. This means they are able to form relatively loose and reversible molecular aggregates with a specific geometry. The protein structure is especially important for film formation since it determines the ability of proteins to interact with themselves and other components [30,31].

The reactivity of the individual amino acid side chains plays a major role for protein modification. Amongst others, glutamic and aspartic acids as well as lysine are very important. Glutamic and aspartic acids are important because of their carboxyl groups and lysine because of its amino group. The reactivity of these side chains depends on the different reagents and also on their positions within the protein structure and on the pH [32].

### 2.2. Characteristics of Protein-Based Films and Coatings

A film is a preformed thin layer which can be placed around or between foods, while a coating is directly formed on food products as a coat [33,34]. In order to compare the effects of various treatments on protein films, their mechanical and barrier properties have to be determined. The mechanical characteristics described in the relevant literature are as follows: (a) the tensile strength (TS) which is the pulling force per film cross-sectional area required to break the film; (b) the elongation at break for the degree to which the film can stretch before breaking, and (c) the elastic modulus or Young’s modulus which provides information about a film’s resistance to deformation. As for the barrier properties, the water vapor permeability (WVP) along with relative humidity (RH) and the oxygen permeability (OP) are considered [35,36,37,38].

### 2.3. Processing of Protein-Based Films and Coatings

There are two technological processes used to make these protein-based materials, namely the “solvent process” and the “thermoplastic process” [39]. During the solvent process, the proteins are dispersed and solubilized in solvents such as water or ethanol, followed by casting, spraying, or dipping (shaping) and then drying. With the aid of plasticizers and rising temperature, proteins pass the glass transition during the thermoplastic process. Consequently, a rubbery mass is created which can be shaped and is stabilized by cooling or by eliminating the volatile plasticizers (e.g., water) [40,41,42]. Thermoplastic processes such as compression molding and extrusion are vital for industrial applications because they allow the profitable production of films and coatings on a larger scale [43,44].

### 2.4. Whey Proteins

There has been a large amount of interest in recent years in the production of whey protein based films and coatings. Whey proteins are a by-product from the precipitation of proteins in milk and during cheese manufacturing [45,46]. Whey is a dilute nutrient stream. It can be dried to provide whey powder. Depending on the protein content, the powder is called either whey protein concentrate (25%–80%, WPC) or whey protein isolate (WPI) which contains >90% protein on a dry weight basis [47].

The major proteins in whey products are α-lactalbumins (α-La), β-lactoglobulins (β-Lg), bovine serum albumin, immunoglobulins, and proteose-peptones. β-Lg is the most prevalent protein in the whey protein fraction. It makes up about 57% of the protein in whey [48]. Monomeric β-lactoglobulins contain one free sulfhydryl group and two disulfide groups [49]. Studies have shown that β-Lg exists in a globular form, with a hydrophobic center which can be involved in the binding of hydrophobic molecules [50]. The second most abundant protein in whey, α-lactalbumin, accounts for about 19% of the total whey protein [48]. It is a globular protein with great flexibility due to approximately 61% unordered secondary structure [51]. Nevertheless, bound calcium is responsible for intermolecular ionic bonding and S–S bridges maintain sufficient stabilization. In addition, disulfide interchange reactions occur with the β-lactoglobulin component [26]. Bovine serum albumin (BSA, 7%) is also a globular protein. It contains 17 disulfide bonds and one free thiol group and is therefore highly relevant for the formation of the films. Due to effective binding of free fatty acids and other lipids, BSA is stabilized against denaturation [52]. Immunoglobulins and proteose-peptones respectively make up to 6% and 11% approximately of total whey proteins [30].

### 2.5. Soy Proteins

Soy protein is extracted from soybeans during the production of soy oil. Soy flour is a secondary product and it can be purified to obtain soy protein isolate (SPI) [53]. Soy protein isolate is a mixture of proteins having different molecular properties. About 90% of soy proteins are globulins [54]. Globulins are protein fractions in which the subunits are associated via hydrophobic and hydrogen bonding [55]. The soy protein globulins can be fractionated into 2S, 7S, 11S, and 15S according to their sedimentation coefficients. The 7S (β-conglycinin) and 11S (glycinin) fractions make up about 37% and 31% of the total extractable proteins and have the ability to polymerize [56,57]. Structural differences mean there are variations in the functional properties of the 7S and 11S fractions [58]. The extensively glycosylated 7S protein consists of three peptide subunits (α, α′, and β) which results in different film formation depending on their combinations [59]. On the other hand, the sulfhydryl groups of the 11S protein were reported to be responsible for the formation of disulfide links which result in the formation of a three dimensional network [54,60].

### 2.6. Wheat Gluten Proteins

Wheat gluten proteins are the storage proteins of wheat. The water insoluble fractions of the wheat proteins are prolamin and glutelin. In wheat, the prolamin fraction is called gliadin and the glutelin proteins are referred to as glutenin. Both represent respectively about 33% and 46% of the total protein content in wheat. Prolamin is considered as the solvent for glutelin and therefore the main factor responsible for the viscosity of gluten. On the other hand, the fibrous glutelin fraction in gluten defines its elasticity and firmness. The glutelin fraction can be divided into two different subfractions, high molecular wheat glutelin (HMW) and low molecular wheat glutelin (LMW). Moreover, the HMW subunit can be separated into two types, x and y. Likewise, the prolamin fraction consists of ω5-, ω1,2-gliadine as well as α- and γ-gliadin. The content of subunits varies with the wheat variety. According to the molecular mass of the gliadin and glutenin subunits, three distinct groups of proteins can be defined: high molecular mass, medium molecular mass, and low molecular mass. These groups are shown in Table 1 with their wheat gluten and cysteine amino acid contents [61,62,63].

## 3. Physical Modifications of Protein-Based Films and Coatings

### 3.1. Heating

It is well known that proteins usually tend to irreversibly aggregate and eventually form gel networks when exposed to high temperatures. Increasing the temperature results in greater mobility of the peptide chains, so changing the protein structure and aggregation state. This leads to different inter and intramolecular hydrophobic and electrostatic interactions as well as hydrogen and disulfide bonds due to the presence of cysteine residues. The properties of the resulting protein network strongly depend on the temperature and also on the ionic strength and the presence of other molecules [21,64,65]. In the literature this treatment is often described as heat curing which is, according to Soroka [66], a process where a substrate is exposed to one or more heating cycles aimed at changing the molecular structure and rearranging the polymers.

Thermal denaturation of whey proteins begins at approximately 70 °C [67], whilst for gluten proteins it starts at 90 °C [68] and for soy proteins at 90 °C [69]. However, the denaturation temperature strongly depends on the protein concentration and the solvent properties [70]. For example, in an extrusion process the soy protein denaturation temperature was found to be 120 °C [71].

The thermal pretreatment of proteins, and also heating a formed protein film, leads to augmented crosslinking of the disulphid, hydrogen, and hydrophobic bonding types. Thus, whey, soy, and gluten based films processed under increased temperature show a significantly higher tensile strength [17,23,27,72,73,74,75,76,77,78,79,80,81,82].

Vachon et al. [77] ascertained that pretreated WPC-based films are weaker than WPIs. Perez-Gago [79] reported a significant lower oxygen permeability after heat treatment of whey protein films. Heat treatments also involving a reduction of the water content increase the film’s strength and rigidity [80,81]. Barreto et al. [83] investigated the thermal denaturation of WPC films and reported two denaturation stages. The first was due to water loss up to 200 °C and the second involved degradation starting at 295 °C. The thermal stability was highly dependent on other molecules in the film.

Heat treatment of soy protein prior to film formation produces smoother and more transparent films with reduced water vapor permeability [84]. Furthermore, the heat curing of soy and gluten protein films increases their elongation properties [23,78,84].

### 3.2. Shearing

Shearing can have three distinct effects on proteins: aggregation, de-aggregation, and protein denaturation. However, extremely high shear rates are necessary to denature proteins [85].

The breakup of aggregates is due to erosion of the particle surface and pressure changes in the fluid leading to deformation and fragmentation of the particles [86,87]. On the other hand, an increased collision rate can favor the formation of aggregates. The collision of small particles (below 1 µm) is driven by Brownian motion while larger particles are influenced by the fluid flow induced by shear. The presence of larger and more numerous protein aggregates therefore increase the number of collisions during shear treatment [88,89].

Steventon et al. [86] reported a break up of whey protein aggregates during the shear treatment and short period of heating in an extrusion process. The opposite was observed by Cheftel et al. [90] for whey and soy protein while processing with longer heating time. More recently, Wolz et al. [91] ascertained that increasing the whey protein concentration up to 30% results in smaller, more compact, and stable aggregates. This is due to the higher viscosity and shear stress. The particle size increases at first at low protein concentrations due to higher collision rates. The higher shear stress occurring with increased protein concentration leads to the formation of smaller particles. Fang et al. [92] reported that mechanical shearing during the extrusion of soy proteins also reduced the size of its particles.

Pommet and Redl et al. [93] stated that high shear rates significantly lower the activation energy for the crosslinking of gluten proteins.

### 3.3. Hydrostatic Pressure

#### 3.3.1. Mechanism and Effects of Hydrostatic Pressure

Hydrostatic pressure (HP) processing uses water as a medium to transmit pressure from 100 to 1000 MPa to foods under isothermal conditions [94,95,96,97]. The effect of pressure on proteins is often described by the principle of Le Chatelier, whereby a system reduces its free energy by minimizing the effect of the external factor. Consequently, a change in pressure is compensated by modification of the system volume [98,99]. In solution, the volume of a protein is determined by the volume of its atoms and the internal cavities due to imperfect packaging of the amino acid residues. On the other hand, a volume decrease results from peptide bonds and polar amino acid hydration [100]. Therefore, almost all globular proteins have a positive compressibility due to their cavities [101].

HP thus disrupts intermolecular hydrophobic and electrostatic interactions whose formation result in a volume increase. It also appears that HP increases the reactivity of sulfhydryl groups. However, high pressure stabilizes hydrogen bonds since their formation slightly reduces the volume. Covalent bonds are not affected. Consequently, high hydrostatic pressure changes the quaternary, tertiary, and secondary conformations of proteins as well as their ionic and hydrophobic stabilized aggregates but has no influence on the primary structure. Proteins subsequently unfold and, if their concentrations are high enough, form gel networks and precipitate [102,103,104,105,106,107].

Hydrophobic interactions are strengthened or weakened depending on the protein itself, its state in solution, and other unsettled influences [108,109]. Hence, the behavior of proteins under pressure cannot by determined from known structural models and thus require individual testing [110].

#### 3.3.2. Physical Influence of HP on Whey, Soy, and Gluten Protein Films and Gels

Up until now only a few studies on the influence of HP processing on protein-based films and coatings have been published [19,111]. A patent has been granted for using this process to produce films [112]. This section therefore mainly summarizes the effects of HP on whey, soy, and WG protein gels used to produce edible films and coatings.

High pressure affects the conformation of whey proteins and dissociates large aggregates, exposing hydrophobic groups by unfolding and allowing the formation of intermolecular sulfhydryl bonds [104,113,114,115,116,117,118,119]. Compared to thermal processing, pressure induced β-Lg gels appear to form a more porous and thicker stranded structure with weaker intermolecular interactions. The resulting gels have stronger water exudation and water solubility, lower rigidity, and the proteins tend to aggregate during storage [116]. Increased pressure leads to harder gels with higher breaking stress and lower solubility [120,121]. However, van Camp et al. [122] produced WPC gels with comparable strength to those induced by heat and even stronger with high protein concentration. Gentle thermal pretreatment (55 °C) of whey protein improves film forming ability but higher temperatures have a negative effect due to protein aggregation [123].

The formation of high pressure induced soy protein gels has also been reported. The gels had lower hardness than the ones produced by thermal treatment and had high water holding capacity [124,125]. Speroni et al. [126] showed that β-conglycinin and glycinin formed hydrophobic interactions and disulfide bonds during HP processing. However, HP induced weak gels with low elasticity and diminished the ability to form hydrophobic interactions on heating. Increasing pressure and holding time leads to a stronger soy protein gel network with stronger hydrophobicity possibly crosslinked by disulfide or hydrogen bonds [127,128,129]. The formation of random coiled structures from β-sheets and α-helices by HP was observed [130]. Subirade et al. [131] indicated that β-structures might be essential for the formation of soy protein film networks.

Low pressure processing (200 MPa) of WG results in an increase in the ethanol soluble fraction and thiol content. It seems that the α- and γ-gliadins in the cysteine are sensitive to pressure while the ω-gliadins only change their conformation and transfer to the ethanol soluble fraction. The glutenin fractions, which are relatively rich in thiol groups, are also strongly affected by high pressure. Higher pressure and temperature cause significant gluten strengthening allowing the production of stronger gels than solely by heat treatment [116,122,132]. Apichartsrangkoon [133] stated that significant S–S crosslinking only occurs under extreme conditions (800 MPa for 50 min).

In a gluten-soy blend gel, the large gluten proteins showed much greater gel strengthening influenced by higher concentration, pressure, and temperature than the smaller soy proteins [134].

### 3.4. Ultrasound

#### 3.4.1. Mechanism and Effects of Ultrasound

Ultrasound is defined as an acoustic wave with a frequency higher than 20 kHz, which is the upper threshold of human auditory detection [135]. It can by differentiated into two categories which are described differently depending on the author and application: Low and high energy, low and high power, low and high intensity, and so on. Low energy ultrasound ranges from 100 kHz to 1 MHz at intensities lower than 1 W·cm^−2^ while high energy ultrasound ranges from 16 to 100 kHz at intensities over 1 W·cm^−2^ [136,137,138,139].

High intensity ultrasound in a liquid generates cavitation due to the compression and decompression cycles of the sonic waves. The violent collapse of the gas bubbles results in high shearing effects. These shear forces and the energy inputs are strong enough to break covalent bonds of proteins dissolved in aqueous solutions [140,141]. Furthermore, regions of high local pressure and temperature up to 50,000 kPa and 5000 K are induced [142,143].

Due to the thermal decomposition of water, free hydroxyl and hydrogen radicals are formed [144,145]. This sonolysis of water, along with the shear forces, is mainly responsible for the denaturation of proteins by high intensity ultrasound [146,147]. The high temperatures are thought to have a negligible effect on biotechnological processes due to the narrow ranges. However, the gas bubbles generate microstreaming favoring the convection of reactive components. Consequently, chemical reactions taking place in proteins are accelerated. Thus, the turbulence created by ultra-sonication can also be used for homogenization. The strength of these effects is strongly dependent on the wave characteristics and distance from the emitting electrodes [148,149].

#### 3.4.2. Influence of Ultrasonic Processing on Protein-Based Films and Coatings

Kadam et al. [150] investigated the effect of ultrasound on whey protein isolate films containing nanoparticles. Before casting, the WPI solutions were sonicated at increasing amplitudes to obtain a homogenous protein-based film. The results showed there was a significant increase in film strength, elasticity, and hydrophobicity with higher sonication amplitude. This was stated as being due to improvement of the homogenous distribution of the film by sonication. However, the water vapor permeability remained unchanged. The same results had previously been obtained by Barnerjee et al. [151]. This could be explained by the smaller particles in film forming solutions obtained by the sonication process. The increased molecular interactions due to the energy input could lead to higher molecular order and therefore increased film strength [15]. Chen et al. [152] reported that intense ultrasonic treatment of whey drives off more moisture from the protein films. This could not be confirmed by Barnerjee and Kadam who observed no significant changes in the moisture content.

Rodriguez et al. [153] investigated the effect of ultrasound treated whey protein coating on the quality of frozen fish. The sonicated coatings exhibited considerably lower lipid oxidation than the untreated coatings and no sensory changes could be detected. Although protein films generally display a good O_2_ barrier [154], chemical mechanisms could not been excluded.

Furthermore, Guzey et al. [155] determined that high-intensity ultrasonic processing of BSA increases its intramolecular mobility and surface activity.

Jambrak et al. [156] observed an increased solubility and specific surface area in soy protein treated by low-frequency ultrasound. These changes were partially explained by free hydroxyl radical formation. The same explanation was given by Wang et al. [157] as the vapor and oxygen barrier of a soy protein containing film was ameliorated by this process. The same main author also observed increased hydrophobicity in another soy protein containing film treated by ultrasound and attributed this to the cavitation improving the film density [158]. Furthermore, Hu et al. [159] ascertained that the free sulfhydryl content, surface hydrophobicity, and protein solubility of soy protein solutions increased with low-frequency ultrasonic treatment. The pretreatment also bestowed better water holding capacity and gel strength but no changes in the particle size distribution.

Ultrasonic treatment dissolved gluten protein aggregates prior to gluten protein film formation and therefore changed its appearance. Nonetheless, the expected solubility change from the dissolution could not been measured [160,161].

### 3.5. Ultraviolet and γ Irradiation

Radiation can be absorbed by atoms and molecules in a system. The captured energy is converted into chemical energy, inducing photoisomerization. Consequently, the exposure of double bonds and aromatic rings of proteins to UV radiation (180–400 nm) lead to free radical formation in amino acids such as tyrosine and phenylalanine. These changes induce the formation of intermolecular covalent bonds [162,163]. Ionizing radiation such as γ and high-frequency UV radiation additionally causes oxidation of amino acids, rupture of covalent bonds, formation of protein free radicals, and water radiolysis producing free oxygen radicals. Brault et al. [164] demonstrated that γ-irradiation induces the formation of bityrosine bridges in caseinate-based films. Irradiation therefore can have a direct influence on proteins as well as an indirect influence by impairing the surroundings [162,165,166,167,168,169,170,171,172].

#### 3.5.1. Effect of Ultraviolet Irradiation on Protein-Based Films and Coatings

Ustunol et al. [173] observed a significant increase in the tensile strength of whey-based films by UV irradiation of the protein solution prior to film formation. However, the radiation treatment had no influence on the barrier properties and elongation at break of the films. The same effects were reported by Schmid et al. [13] who investigated the impact of UV irradiation on cast whey protein films. The German research team also concluded that increasing the radiation dose leads to increased molecular interactions within the protein network.

Gennadios et al. [162] reported a linear increase in the tensile strength and a linear decrease in the elongation at break with increasing UV treatment intensity (λ = 253.7 nm up to 103.7 J·m^−2^) of cast soy protein films. These results could not be confirmed by Vaz et al. [174] who treated the soy protein solution and the films at a wavelength of λ = 366 nm and 4.5 J·m^−2^. This research team only observed a small increase in the mechanical properties of the films with no change in the protein network.

An increase in tensile strength was ascertained by Rhim et al. [163] after UV irradiation (253.7 nm, 51.8 J·m^−2^) of cast wheat gluten protein sheets. However, Micard et al. [27] reported no significant change in the mechanical properties after UV treatment (λ = 254 nm and 1 J·m^−2^) of films based on the same protein.

Furthermore, increased yellowness of whey, soy, and wheat gluten films has been observed after UV irradiation [13,162,163].

#### 3.5.2. Effect of γ Irradiation on Protein-Based Films and Coatings

γ irradiation (32 kGy) of WPI solutions increases the content of β-strands and β-sheets. This could explain the finer stranded network and increased fracture strength of the whey protein gels and films cast with the irradiated solutions [175]. Cho et al. [169] reported that γ-irradiation of BSA and β-Lg in solutions caused the disruption and degradation of the protein structure as well as crosslinking and aggregation of the polypeptide chains. Furthermore, Ouattara et al. [176] detected a significant decrease in the water vapor permeability and an increase in the molecular weight of the protein particles in solution after irradiation using the same dose.

Lacroix et al. [177] investigated the effect of γ irradiation prior to casting SPI and SPI/WPI blend films. There was an increase in the puncture strength of both films after radiation treatment at 128 kGy. However, there was only a reduction in the water vapor permeability for the SPI film, while the blend film remained unchanged. Sabato et al. [60] also reported an increase in puncture strength and puncture deformation after γ-irradiation (32 kGy) of SPI and SPI/WPI films. Here, the effect was also greater on the SPI film.

Micard et al. [27] reported an increase in TS and WVP as well as a decrease in elongation for γ-irradiated gluten films (10 kGy). Higher radiation doses up to 40 kGy had the inverse effect. This could be explained by the decrease in glutenin solubility by breaking down covalent linkages and de-polymerization [164,178].

The effect of γ-irradiation on SPI and gluten solutions prior to film formation was investigated by Lee et al. [179]. The viscosity of the gluten-based films decreased at a radiation dose below 16 kGy due to cleavage but increased at higher dose up to 50 kGy due to protein aggregation. As for the SPI-based films, only a decrease in viscosity was reported. Furthermore, the WVP decreased by up to 13% for the SPI-based film and by 29% for the gluten-based film while the TS increased by respectively 2- and 1.5-fold at 50 kGy.

### 3.6. Thermoplastic Processing

**Figure 1:**
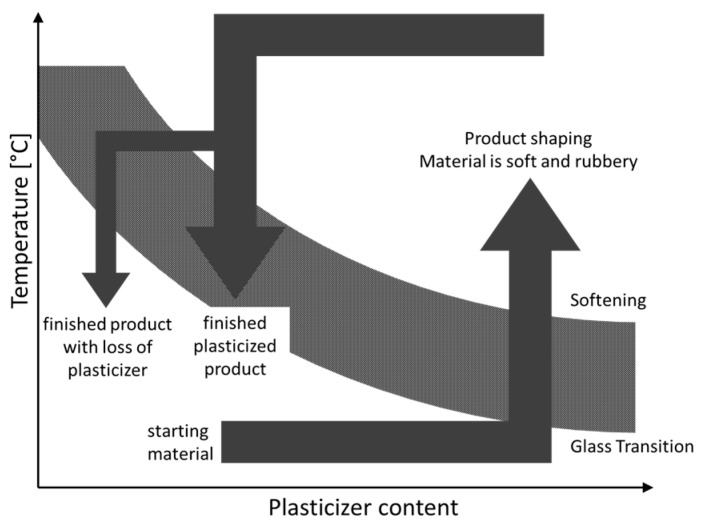
Effect of plasticizer content on thermoplastic processing, adapted from [4].

Increasing the chain mobility by denaturation of the film forming proteins is essential for thermoplastic processing. Changes in chain mobility are related to thermal transitions such as the glass transition temperature (T_g_) and flow temperature (T_f_), also referred to as the softening temperature, which occurs at higher temperature. Above the T_f_ the protein-based mixture exhibits the low viscosity necessary to process the material. Unfortunately, proteins with less than 5% water have a T_g_ above or equal to their decomposition temperature [180]. The temperature difference between the T_g_ and T_f_ of soy and gluten based films is about 40 °C [181]. Moreover, films based solely on proteins are fragile and brittle because of the bonds and interactions between the protein chains [182]. As shown in Figure 1, the addition of plasticizers resolves this issue by impairing macromolecular associations and therefore reducing the T_g_ and T_f_. The chain mobility increases on addition of plasticizers, due to replacement of protein interactions with protein-plasticizer interactions [183].

Water is the most effective plasticizer but it increases the melt viscosity at higher content [180]. This leads to reduced protein transformation resulting from the small temperature increase due to the small motor torque and specific mechanical input [184]. Therefore, other plasticizers varying in size, shape, composition, and hydrophilicity are used depending on the protein source and the use of the thermoplastic product [185]. As an example, glycerol inserts itself within the polymer network [186] and is therefore an excellent plasticizer widely used for the thermoplastic processing of proteins [82,93,184,187,188,189,190,191].

#### 3.6.1. Compression Molding

In compression molding, protein-plasticizer mixtures are placed in an open mold. Subsequently, a plunger applies pressure forcing the material to assume the desired form [192]. This usually occurs relatively quickly at low moisture contents, high temperature, and pressure in order to transform the blends into viscoelastic melts. The combination of heat and pressure leads to the denaturation of the proteins [193]. The subsequent cooling of the product determines its form by covalent, ionic, hydrogen bounding as well as hydrophobic and hydrophilic interactions [108]. Knowledge about optimal ingredient ratio and processing parameters are also vital for film formation by extrusion [82,191].

Sothornovit et al. [191] formed transparent WPI-based films by compression molding with 30% to 50% water or glycerol. The films processed with water were brittle, while those containing glycerol as plasticizer were flexible. The same research team also investigated the effects of the plasticizer content and processing conditions compared to casting. The processing pressure and temperature had a negligible effect on the mechanical properties of the protein sheets produced at up to 140 °C and 2.25 MPa for 2 min. However, the tensile strength and elastic modulus were smaller at higher glycerol content [82].

Soy protein films molded under compression at high temperatures up to 150 °C with glycerol usually show higher elongation and tensile strength than the ones obtained by conventional casting [193,194,195,196]. The soy protein-based films produced by thermoplastic processing are also more transparent [53,196]. Ciannamea et al. [196] reported lower WVP but higher OP for the soy protein films. The same research group also ascertained that disulfide bonds were mainly responsible for the characteristics of the compression molded films whereas hydrogen bridges and hydrophobic interactions dominate in the cast soy protein films.

Increasing the processing temperature during compression molding leads to higher crosslinking of the wheat gluten protein network. This results in improved tensile strength and lower WVP and OP [197,198]. The activation energy for the crosslinking was found to be 170 kJ/mol by Pommet et al. [93]. However, longer holding times seem to have a smaller effect than the temperature [43].

#### 3.6.2. Extrusion

Up until now, extrusion is the most commonly used process for polymer production [199]. It is a continuous process whereby the raw materials are constantly introduced into a hopper feeding a horizontal barrel. The extrudate is subsequently conveyed by one or two rotating augers and finally pushed through a die. This process can involve various operations such as heating, shearing, mixing, compressing, melting, and shaping. Generally, the extruder barrel is composed of three sections: the feeding, transition, and metering section. The ingredients are introduced into the feeding section where also mixing, degassing, and slight compression occur. The continuous rotation of the screw(s) also moves the mixture to the transition zone. As indicated by the name of this section, this is where the raw material changes into extrudate whilst the pressure and temperature increase. This effect is induced by reduction of the flow channels, so compressing the product and dissipating mechanical energy. In the subsequent metering section, also referred to as the heating or cooking zone, the highest temperatures, pressures, and shear rates are experienced. This is also where the product acquires its final aspect before it is pressed through the die [184,200,201].

The extrusion process can proceed under various conditions using different screw configurations, lengths, diameters, speeds, temperature profiles, feeding rates, and the addition of ingredients at the beginning and during the process [184].

Onwulata et al. [202] extruded a WPC mixture containing 38% moisture with a twin screw extruder at temperatures ranging from 35 to 100 °C. Under these conditions the degree of denaturation of the proteins increased from 30% to 95%. The processed proteins where subsequently dried, mixed with water, and then heated to form gel samples. The gels obtained from proteins extruded at the lowest temperatures up to 45 °C were stronger than the ones produced with unprocessed WPI. However, the gels made with proteins processed at higher temperatures were weaker. Hernandez et al. [184] investigated the effect of glycerol and moisture content on the thermal transitions of WPI during extrusion. There was a decrease in TS and elasticity with increasing glycerol content. The same main author also investigated the heat seal strength of extruded whey protein films [203]. In this study, extruded sheets showed lower seal strength then cast sheets since they were thicker and therefore harder to seal. In a study conducted by Qi and Onwulata [204] higher moisture content slightly increased the protein solubility and reduced the content of β-Lg while the α-La remained unchanged. Schmid et al. [41,42] extruded WPI with ethylene vinyl acetate (EVA). The resulting sheets exhibited higher WVP with increasing WPI content compared to pure EVA films.

Zhang et al. [205] investigated the mechanical and thermal properties of soy protein films extruded with various plasticizers. The film sheets were relatively strong and elastic except the ones with high moisture and glycerol content. It was ascertained by Chen et al. [206] that non-covalent bonds were more important for the soy’s extrudate structure than covalent bonds.

## 4. Chemical Modifications of Protein-Based Films and Coatings

This section deals with chemical modifications. This includes reactions with chemical agents and modification by pH alteration. For these modifications the protein side chains play a major role. Table 2 shows the reactive groups of the side chains and their occurrence in selected proteins. In addition to the side chain composition, the location within the protein and the external conditions such as the pH also influence the reaction rates [32,207].

### 4.1. Reactions with Chemical Agents

#### 4.1.1. Alkylation

**Figure 2:**
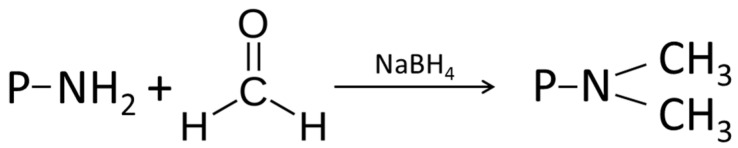
Reductive methylation, adapted from [207].

The substitution or addition of alkyl groups in organic compounds is called alkylation [207]. The alkylation of proteins mainly takes place at the amino groups of protein side chains. Therefore lysine is essential for this reaction. For example, the addition of formaldehyde in combination with sodium borohydride to the film building solution leads to reductive methylation (Figure 2). The reaction starts with the condensation of the amino group by a carboxyl group, resulting in the formation of an imine. The imine is subsequently reduced by a mild reducing agent, such as sodium borohydride, which is oxidized itself at the same time. Thereby methylamino groups form. These are immediately transformed into dimethylamino groups by additional formaldehyde and reducing agent, whereby formaldehyde acts as the oxidant. The formation of these dimethylamino groups, which replace the initially amino groups, is responsible for the change in the functional properties of the proteins [32]. The reaction is illustrated below.

Reductive alkylation is not a commonly used method for improving the functionality of proteins. However, some studies have demonstrated slight modifications. For example, Kester et al. confirmed changes to the functional properties of proteins [207]. However, the effects of this chemical modification are so slight that reductive alkylation was used to perform studies relating to the protein structure, such as radiolabeling. In order to significantly enhance the functional properties of proteins, formaldehyde has to be replaced by carboxyl compounds with a more complex structure. For example, propylenglycol alginate, with sodium cyanoborohydrid as a reducing agent, is used in soy protein films to improve the mechanical properties [209].

#### 4.1.2. Acylation

For an acylation reaction a protein must have a nucleophile amino acid residue, for example an amino or phenol group [207,210]. Such residues react with acylating agents which possess a carbonyl group, such as activated acid anhydrides [30,210]. The acylation of these groups leads to enhanced functional properties, especially in food proteins [211,212,213].

#### 4.1.3. Acetylation

Acetylation, a specific form of acylation, is the integration of an acetyl group into compounds which contain amino, hydroxyl, or thiol groups. In order to accelerate the reaction, acid or basic catalysts, such as zinc chloride or sulfuric acid, can be added [214].

**Figure 3:**
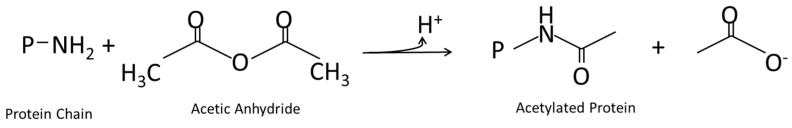
Acetylation of protein chains, adapted from [215].

Acetic anhydrides are used for the acetylation of proteins (Figure 3). This leads to covalent bonding of acetyl groups with the amino groups of the protein. Due to the fact that oppositely charged amino acid side chains attract each other, a reduction in the number of amino groups leads to partial unfolding of the protein backbone [207] Thereby, for example, the gel strength and water binding capacity of soy protein isolate (SPI) decrease. The solubility in the pH range from 4.5 to 7 becomes greater [216]. However, Franzen et al. [217] found that acetylation only has a minor effect on the functional properties of soy protein. Compared to the effect of succinylation, the effect of acetylation on the protein structure is significantly smaller [207]. Additionally, Ghorpade et al. [218] showed that acylation with acetic anhydride could not improve the tensile strength nor the water vapor permeation (WVP) or oxygen permeation (OP) of soy protein films.

#### 4.1.4. Succinylation

**Figure 4:**
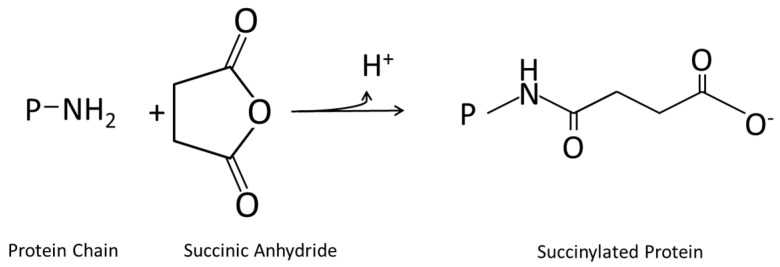
Succinylation of protein chains, adapted from [215].

Succinylation is also a specific form of the acylation reaction. The acylation of amino acid residues in the succinylation reaction is performed with dicarboxylic acid anhydrides, for example succinic acid anhydride [217]. Thereby, H_2_O, which results from combination of a hydroxyl group of the carbonyl group of the acid anhydride with a hydrogen atom of the functional groups of the amino acids, is split off [30]. This reaction is shown in the Figure 4 below.

In general, succinylation leads to improved solubility of the proteins [30]. The acylation mainly occurs at the ε-amino group of lysine. Thereby, the cationic amino group is transformed into an anionic residue. This leads to a change in the foaming properties as well as in the emulsifying capacity. Increased aqueous solubility is a further consequence of the raised net negative charge induced by succinate anions [217]. Barber et al. [219] confirmed this for wheat gluten. Their research showed that succinylation of wheat gluten is able to increase the solubility, emulsification capacity, water adsorption capacity, and water holding capacity. Also, glycinin with 25% succinylation was found to have an increased surface pressure as well as increased film yield stress, film elasticity, and foam stability [220]. In contrast, soy protein isolate films which were succinylated showed neither improved mechanical properties nor improved barrier properties compared to the non-succinylated films. However, they showed enhanced water solubility [109].

#### 4.1.5. Incorporation of Fatty Acid Chlorides (Grafting)

**Figure 5:**
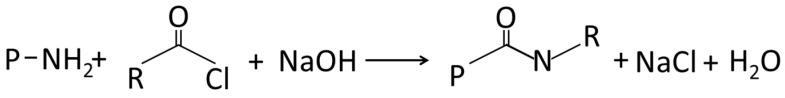
Reaction of proteins with fatty acids, adapted from [225].

The incorporation of fatty acid chlorides represents an acylation reaction. Palmitic acid chloride is commonly used [221]. The process is mainly based on Schotten-Baumann’s reaction [222,223]. For incorporation into protein structures an alkaline medium is recommended. The reaction equation is shown below (Figure 5) [224].

This leads to the integration of long alkyl chains which act as internal plasticizers. These are able to reduce or even interrupt intermolecular interactions between the protein side chains. This causes changes to the thermal properties and protein folding. An important factor is the number of available functional groups, because they determine the number of bonded fatty acid chlorides. For example, Bräuer et al. [221] was able to integrate 4 mmol·g^−1^ palmitoyl groups into wheat gluten as well as into soy protein. Incorporation into corn zein and pea protein was significantly lower. Another factor which influences the substitution of ε-amino groups with fatty acids is the solvent. The dilution of, for example, β-lactoglobulin in an organic solvent showed higher incorporation rates than under aqueous conditions [226]. The acylation has various effects on the functional properties of the protein. First of all, the modified proteins show higher hydrophobicity and therefore higher water resistance. This leads to lower water absorption and the proteins are less hygroscopic, which contributes to poorer welling. Another positive effect concerns the thermal properties. The modified proteins, for example the acylated palmitoyl derivatives, melt between 150 °C and 200 °C and therefore show better thermal characteristics than the native proteins, which cannot be melted. This can be due to the reduction in the intermolecular interactions, especially hydrogen bonding, induced by the modification with fatty acid chlorides [221]. The incorporation of fatty acids is able to improve the moisture barrier of protein films and coatings [29].

Considering all the reactions with chemical agents, it is clear that neither alkylation nor acylation methods are able to improve the technofunctional properties significantly. However, the incorporation of fatty acids is a promising acylation method, although very little research has been carried out to date on this. A big problem with alkylated films is the mostly toxic reagents which lead to non-edible films [209,218].

### 4.2. Modification by pH Alteration

#### 4.2.1. Hydrolysis

**Figure 6:**
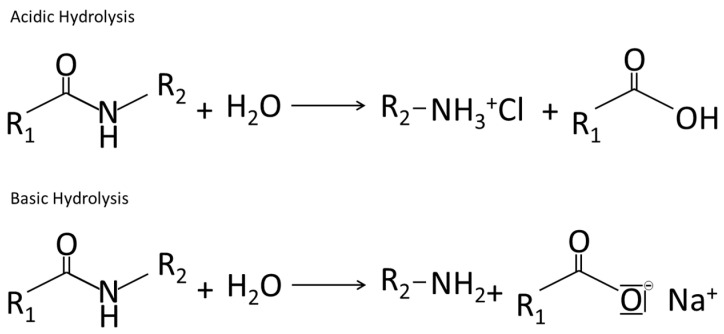
Acidic and Basic Hydrolysis of Proteins, adapted from [30,229].

Little research has hitherto been devoted to the non-enzymatic hydrolysis of food proteins. Acidic and basic hydrolysis of proteins is mainly used to determine the amino acid structure [227,228]. The mechanisms involved here are shown in the Figure 6 below.

Akkermans et al. [230] found that β-lactoglobulin which was heated for 20 h at 85 °C at pH 2 leads to hydrolyzed peptides with molecular masses between 2000 and 8000 Da. Similar studies showed that the hydrolysis influences the viscosity. Mudgal et al. (2011) found that β-lactoglobulin dispersions heated at pH 2 were very viscous. The power law index was about 0.33 at 7% (*w*/*w*) and about 0.17 at 8% (*w*/*w*). This means that the solutions demonstrate pseudoplastic behavior (7% (*w*/*w*)) or even form solid like gels (8% (*w*/*w*)) [231]. All in all, the effects of acidic and basic hydrolysis on the functional properties of proteins should be similar to the effects of enzymatic hydrolysis due to the fact that both reactions shorten the protein backbone by splitting peptide bonds.

#### 4.2.2. Change in Protein Structure

An important factor for the functional properties of proteins is the pH value. This is due to the fact that proteins change their net charge at a pH value that is different to their isoelectric point. There is a positive net charge at a lower pH value and a negative net charge at a higher pH value [29].

In SPI and wheat gluten films, intermolecular interactions such as disulfide bridges, hydrogen bonds, and hydrophobic interactions are especially influenced by a pH shift. SPI films in the pH ranges 1–3 and 6–12 showed the best film formation properties while homogenous free standing WG films were achieved in the pH ranges 2–4 and 9–13. Between those ranges, more precisely pH 4–5 for SPI and pH 5–8 for WG, there was rather poor film formation or even no film formation at all. These ranges more or less correspond to the isoelectric point, which is pH 4.5 for SPI and 7.5 for wheat gluten. The main components of wheat gluten, gliadin and glutenin, have isoelectric points at 8.1 and 7.1 respectively [232,233,234]. The SPI films which were produced at an alkaline pH (6 to 11) had higher tensile strength, higher elongation at break, and lower water vapor permeability than the films produced under acidic conditions. The WG films formed in the pH range from 9 to 13 had significantly higher tensile strength compared to the films produced in a pH range from 2 to 4 [232].

In whey protein films, pH alteration also changes the quaternary structure of β-Lg. In particular, at low temperatures and at low concentration of β-Lg the pH value is responsible for changes to the quaternary structure [234,235].

**Figure 7:**
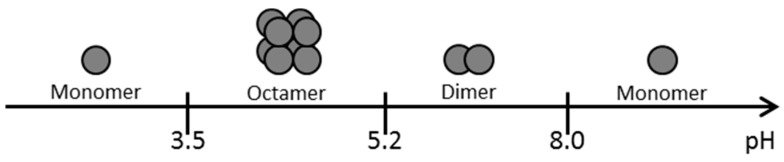
Quaternary structure of β-Lg as a function of pH at low temperature and low concentration, adapted from [234].

As can be seen in Figure 7, a pH value smaller than 3.5 or higher than 8.0 leads to a monomer, while a pH between 3.5 and 5.2 results in an octamer. In the pH range between 5.2 and 8.0, which is particularly important because it includes the pH of milk, β-lactoglobulin forms dimers [234,235].

The effect of pH alteration at pH values between 7 and 9 on the mechanical properties of whey protein films was investigated by Anker et al. (1999). Amongst other things he found that the Elastic Modulus and the stress at break developed a maximum at the critical gel concentration at pH 7 and 8, while the elongation at break increased with rising pH value [236]. Regarding the barrier properties, it can be stated that the hydrophilic whey protein films have a poor water vapor barrier compared to other non-hydrophilic materials. As such the pH value has only a minor impact on the water vapor permeability. In the pH range from 4 to 9, the WVP at pH 5 was slightly increased whilst all the other parameters showed no significant differences. Additionally, it was attempted to form films at pH 3 but the lack of film forming ability prevented this [237].

Li and Lee [238] determined that glutenins and gliadins were mainly responsible for wheat gluten film formation during extrusion. It was also found that during this process the WG proteins primarily aggregate through intermolecular disulfide bonds and hydrophobic interactions. The authors also stated that high temperatures of about 185 °C might disrupt the aggregation forces. As for whey and soy protein films, Hochstetter et al. [239] found that the mechanical properties of extruded WG films decrease with higher moisture content. However, the OP was as low as for cast WG sheets.

All in all, a pH change in the film forming solution influences both the mechanical and barrier properties. As such the isoelectric point of the protein that is used plays an important role. The water vapor permeation and the oxygen permeation of the films have their maxima at the isoelectric point. Furthermore, both WPI and SPI films showed increasing elongation at break with increasing pH. Comparing the elongation of SPI films with WPI films during pH alteration, it is apparent that the WPI films are able to double their values, while the levels of the SPI films are between about 70% and 90% [232,236,237].

## 5. Biochemical Modifications of Protein-based Films and Coatings

### 5.1. Biochemical Transformation by Enzymes

Enzymes are proteins having catalytic activity which are synthesized by organic cells. They are able to influence a high number of reactions in proteins, for example hydrolytic reactions such as the splitting of peptide bonds, transfer reactions, redox reactions, and crosslinking. Enzymes are sensitive to pH alteration and high temperatures. They reduce the activation energy of otherwise non-spontaneous reactions and enhance the reaction speed. Enzymes are generally specific to the substrates of the catalyzed reaction or to the reaction itself [30].

#### 5.1.1. Enzymatic Hydrolysis

**Figure 8:**
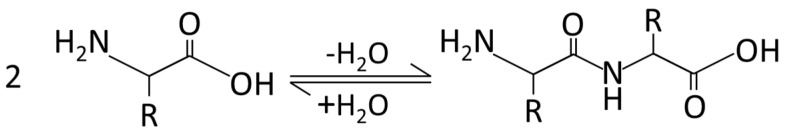
Protein hydrolysis and synthesis of peptide bonds [30].

Proteins are formed by acid-amino-bonds, also called peptide bonds, which link the amino acids together. Hydrolysis leads to the cleavage of these bonds, whereby the proteins end up again as amino acids (Figure 8) [30].

Partial enzymatic hydrolysis is able to improve the solubility of many proteins. Enzymes which are specific for the hydrolytic splitting of peptide bonds are called peptide hydrolases or peptidases. There are two types of peptidases. Exopeptidases catalyze the cleavage of terminal peptide bonds and thus gradually separate single amino acids or dipeptides. Endopeptidases cleave the peptide bonds of nonterminal amino acids [30].

The shortening of the protein chains and consequent reduction of the molecular weight can lead to reduced intermolecular interactions between the individual protein chains. This contributes to higher flexibility of the molecule chains and increased free volume [185,240].

The degree of hydrolysis (DH) represents the amount of hydrolyzed peptide bonds. In particular it indicates the percentage of cleaved peptide bonds relative to the total number of peptide bonds. The higher the degree of hydrolysis the smaller the protein fragments [241]. The influence of the degree of hydrolysis on the mechanical properties and oxygen permeability in whey protein coatings was previously analyzed by Sothornvit and Krochta [185]. They found that the application of proteases to reduce the molecular weight of the protein chains led to an increase in the flexibility at constant oxygen permeability. Whey protein films with the required mechanical properties and adequate oxygen permeability could be cast using a decreased amount of plasticizer. Later Schmid et al. [242] performed a study with a constant amount of plasticizer and increasing concentrations of hydrolyzed whey protein. It was demonstrated that the barrier properties remained almost at the same level, while the tensile strength and Young’s modulus decreased. The enhancement of the functional properties of soy protein isolate by enzymatic hydrolysis has been cited in many publications [243,244,245,246,247]. Unfortunately, an increasing degree of hydrolysation leads to decreased mechanical properties, while the barrier properties almost remain the same [185,227,228].

#### 5.1.2. Crosslinking by Transglutaminase

**Figure 9:**
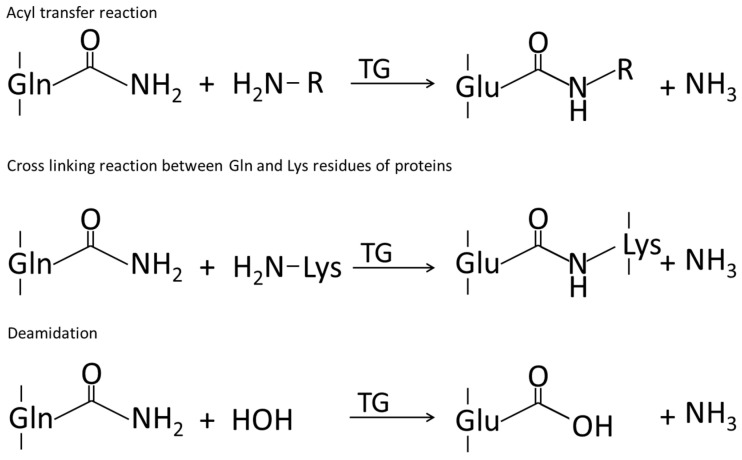
Reactions catalyzed by transglutaminase, adapted from [30].

Transglutaminases are enzymes which catalyze the modification of proteins. They catalyze the acyl transfer reaction between the γ-carboxyl group of a glutamine side chain and the ε-amino group of a lysine side chain. Thereby a ε-(γ-glutamyl) lysine bond is formed between the protein chains [248]. This bond, which crosslinks the protein chains, is stable to proteolysis and improves the enzymatic, chemical, and mechanical stability [249]. However, transglutaminase catalyzes three reactions in total (see Figure 9 below).

Transglutaminase is also used to form biopolymers containing different proteins such as 11S soy protein and whey protein [250,251]. Biopolymers were also formed by the transglutaminase catalyzed crosslinking of soy protein and casein. This was demonstrated by HPLC and SDS-polyacrylamide gel electrophoresis [252,253]. Due to its high number of glutamine side chains, wheat gluten is particularly suitable for protein crosslinking using transglutaminase. Wang et al. [254] also discovered that heating increases the number of ε-(γ-glutamyl) lysine bonds in wheat gluten, due to the increasing number of accessible glutamine and lysine residues [254,255]. In whey proteins, denaturation which results in unfolding of the chains is an important factor for the crosslinking. It is the unfolding which leads to accessible lysine and glutamin residues [256]. Furthermore, in whey protein films the transglutaminase crosslinking reaction increased the average tensile strength [251]. For soy protein isolate films, the functional properties could be improved. Tang et al. [257] reported a decrease in the moisture content and the total soluble matter due to the transglutaminase treatment. The elongation at break was lowered. In addition, the tensile strength and the surface hydrophobicity were increased. However, the water vapor transmission rate and water vapor permeability remained at almost the same level.

A number of research groups have employed enzymatic crosslinking to try to increase the cohesion forces of the proteins within the matrix. Aboumahmoud and Savello [258] reported successful crosslinking of whey proteins with guinea pig liver transglutaminase. In a subsequent study they effectually used TG to covalently crosslink α-lactalbumin and β-lactoglobulin for film formation [259]. Yildirim et al. [260] manufactured a transglutaminase-crosslinked WPI and soybean film which showed good stability. Two years later, Yildirim and Hettiarachchy [251] published another attempt to improve WPI films by crosslinking them with 11S globulin. The research showed that all TG-crosslinked films were more than two times stronger than non-crosslinked films. However, the water vapor permeability (WVP) also significantly increased, making the films unusable for products where moisture levels have to be controlled. On the other hand, there may be food products requiring higher levels of humidity for which these films could be useful. Furthermore, Oh et al. [261] successfully developed TG-crosslinked WPI and casein films with lower WVP, albeit not significantly lower. A very successful crosslinking of chitosan-whey protein edible films by microbial transglutaminase was reported by Di Pierro et al. [262]. The films were found to have enhanced mechanical resistance, reduced deformability, markedly improved oxygen and carbon dioxide barrier properties, and also a lower permeability to water vapor. In another study by Hernàndez-Balada et al. [263], biopolymers produced by treating WPI and blends of gelatin with microbial transglutaminase (MTGase) were reported to have improved strength and stability. A small amount of gelatin was sufficient to realize a dramatic rise in viscosity and higher gel strength. Numerous other reports describe improved properties of transglutaminase-crosslinked whey proteins. The result is an improved gas barrier or lower WVP for WPI-based films [264,265,266,267,268].

As for the treatment of whey proteins, transglutaminase is nowadays the most frequently used enzyme for the crosslinking of soy proteins. Motoki et al. [269] crosslinked milk casein and soybean globulin using transglutaminase. Mariniello et al. [270] prepared pectin and soy flour based films in the presence of TG, resulting in smoother surfaces and higher homogeneity. The films had increased strength and reduced flexibility due to a higher degree of association between the different molecular components. Tang et al. [257] were first to report and evaluate the effects of TG treatment on the properties of basic SPI films. They investigated the effect of microbial transglutaminase (MTGase) treatment on the microstructure of SPI films. Low concentrations of MTGase slowed down the moisture loss rate, which was consistent with the increased surface hydrophobicity of SPI films. Moreover, it was shown that MTGase-treated films had a rougher surface, more homogeneous cross-section, higher tensile strength, and lower elongation at break compared to the controls. However, the WVP was not significantly affected by the treatment and the transparency decreased. Jiang et al. [271] were able to confirm these results, indicating that the improvement of the properties of SPI films by MTGase is largely dependent on many processing parameters, for example the enzyme concentration, the pH of the film forming solution, and the temperature. Treatment at low concentration significantly increased the tensile strength whereas high concentrations of MTGase resulted in lower tensile strength. Gan et al. [272] optimized the SPI gels formed using MTGase by subsequent heat treatment with ribose which induced Maillard crosslinking. The resulting gels were free-standing, with an improved mechanical microstructure due to the higher crosslinking density achieved by the combined crosslinking techniques. MTGase was also reported to improve the functional performance of biodegradable nanocomposite materials such as montmorillonite (MMT) nanoclay intercalated with soy protein [273]. Lastly, Weng and Zheng [274] recently reported the formation of compact film network structures and increased film strength, improved water resistance properties, and better thermal stability when treating gelatin films with transglutaminase in the presence of soy protein isolate. On the whole, however, research work to produce enzymatically improved protein films for packaging materials has not yet managed to develop films having physical properties superior to those of synthetic films [29]. This is why research on SPI composite films has gained in popularity. SPI has been blended with many different polymers to achieve the desired properties [275].

According to Schmid and Hammann [208], wheat gluten film-forming solutions are well suited for enzymatic crosslinking by transglutaminase due to the high number of glutamine residues. There are also other studies on the favorable effects of wheat gluten proteins treated with TG [254,255]. However, there is only one study which discusses the effects of enzymatic treatment on wheat gluten based films. Larré et al. [276] investigated the properties of enzymatically crosslinked, deamidated gluten films. TG was able to introduce covalent bonds into the films, resulting in greater insolubility and increased ability to stretch. However, there was simultaneously reduced surface hydrophobicity.

All in all, crosslinking with the formation of ε-(γ-glutamyl)lysine bonds leads to increased tensile strength in both whey protein isolate and soy protein isolate films. The WPI films are able to increase the tensile strength from ~6 to 13 MPa, while the SPI films only show a small increase [251,257]. These differences may result from the different available side chains but they can also be caused by different enzymatic activities. Regarding the barrier properties, it is apparent that the water vapor permeation of these films almost remains at the same level [251,257].

#### 5.1.3. Peroxydase

An early attempt to enzymatically modify the functional properties of soy protein films was undertaken by Stuchell and Krochta in 1994. No improvement in the water vapor permeability and increased brittleness were reported after treatment with horseradish peroxidase (EC 1.11.1.7). However, the enzyme increased the tensile strength and protein solubility while decreasing the elongation. As horseradish peroxidase catalyzes the oxidation of amino acid side chains, so promoting crosslinks, they concluded that oxidative crosslinking is not sufficiently specific to enhance soy protein films [84].

### 5.2. Composite Films and Addition of Bioactive Compounds

An important trend in recent years has been investigation of novel approaches for further enhancement of the barrier and mechanical properties of protein films. Active and intelligent packaging, or bio-packaging, are aspects which have received much attention. An active packaging is a type of material that extends the shelf life, enhances security, and maintains the product quality by changing its packaging conditions and interacting with the food [275]. In summary, these novel approaches can be of a physical, chemical, or biochemical nature. Generally, these approaches involve modification of the protein structure and/or interactions among protein molecules. Another much investigated approach has involved the creation of blended or composite films [35,277].

Proteins show good matrix as co-polymer in many blended films such as mixtures with polysaccharides. The interaction of proteins and polysaccharides is described in various recent review papers [278,279,280]. The sections below give among other compounds a small description of some of those blends, the reader is invited to look up the cited literature for more detailed information.

#### 5.2.1. Addition of Nanocomposites

A novel upcoming trend is the preparation of WPI films with nanocomposites [29]. For instance, Li et al. [281] highlighted that more than 70% of visible light and more than 90% of UV light can be blocked by composite WPI/biodegradable titanium dioxide (TiO_2_) films. A year earlier, Zhou et al. [282] significantly increased the tensile properties and elastic modulus of WPI films by adding less than 1% (by wt.) of TiO_2_ nanoparticles. However, the moisture barrier properties were decreased and both research groups state that improvements are necessary for new composite film combinations. Zinc (Zn) is an essential micronutrient. Shi et al. [283] fabricated nanocrystalline zinc oxide (ZnO) coated with WPI, thus a nutraceutical agent within a WPI coating. Sothornvit et al. [284] fabricated WPI/nanoclay composite films which had decreased water vapor permeation. Also, the incorporation of the nanoclay Colisite 30B into WPI films exhibited gave a significant bacteriostatic effect against *L. monocytogenes*. Lastly, Oymaci and Altinkaya [285] reported excellent results by blending WPI-based films with zein nanoparticles (ZNP). The water vapor barrier and mechanical properties were improved, suggesting that ZNP/WPI nanocomposites films have great potential for use as biodegradable food packaging materials.

As the case for WPI-based films and coatings, nano-additives and nano-biocomposites are very promising materials for enhancing the functional properties of SPI-based films and coatings and this area has excited a lot of interest [286,287,288]. Improvements due to nanocomposites arise from the strong interactions between the matrices and the nano-reinforcements [289], involving a synergistic effect from combination of the matrix and the reinforcement [290]. In a study by Wang et al. [291], nanocrystalline titanium dioxide (TiO_2_) particles coated with SPI were manufactured. TiO_2_ has been studied extensively as the particles are cheap, photostable, and nontoxic at the recommended safe dosage, [291,292]. SPI/TiO_2_ combinations were found to be bactericidal to *E. coli* and *S. aureus* after the films had been irradiated with UV light at 365 nm for two hours. Another extensively studied bio-nanocomposite is montmorillonite nanoclay (MMT) [29]. Due to its unique structure and properties, this nanoclay has proven to be very effective in improving the mechanical properties of biopolymers by bearing a significant portion of the applied stress [293,294]. This has been confirmed by Kumar et al. [295,296], who reported significant enhancements in the tensile strength, elongation at break, and water vapor permeability compared to regular SPI films and by Jin and Zhong [273] who further treated the film with MTGase. Chen and Zhang (2006) and Echeverria et al. (2014) also reported similar results [297,298]. However, Kumar et al. indicated that substantial improvements in the moisture barrier properties are still required in order to match synthetic materials. Gonzalez et al. [290] developed SPI films reinforced with starch nanocrystals (SNC). The films were transparent, homogeneous, and as the amount of SNC increased the films exhibited lower affinity for water and became more resistant and less extendable. Lastly, Li et al. [299] recently succeeded in enhancing the TS, WVP, and thermal stability of soy protein films with peanut protein nanoparticles.

In addition to whey protein and soy protein films, wheat gluten films have been improved by the incorporation of nanoclays such as montmorillonite (MMT). Tunc et al. [300] prepared WG/MMT nanocomposite films. The presence of MMT led to a different protein network structure, resulting in significant reduction of the water sensitivity. The oxygen and CO_2_ permeabilities remained unchanged, whereas TS slightly increased. Guilherme et al. [301] reported that protein-based nanocomposites consisting of wheat gluten matrix (WG) and MMT exhibited lower WVP, confirming the results of Tunc. With the antimicrobial properties of edible films and coatings pioneering the concept of active packaging, Mascheroni et al. [302] developed an antimicrobial delivery system from film-forming solutions containing wheat gluten as matrix, MMT as structuring agent, and carvacrol as active agent. The results demonstrated there was effective retention and protection of the antimicrobial agent (carvacrol) during the processing stage. Last but not least, MMT was sandwiched between two layers of WG, forming a coating for paperboard. The oxygen barrier was around 25 times better than that of a single layer of WG. Moreover, the water vapor transmission was 6- to 8-fold lower than the uncoated paperboard [303].

#### 5.2.2. Addition of Antimicrobial Materials

WPI films are frequently selected as model edible coating materials for incorporating various additives such as antimicrobial agents, hydrophobic materials, and antioxidants [304].

For example, the design and manufacture of edible films with encapsulated antimicrobial materials are a highly promising strategy for advancing active packaging technology, since prior studies have demonstrated the effectiveness of antimicrobial films in reducing the growth of inoculated bacteria [305]. Joerger et al. [305] and Rocha et al. [306] published detailed reviews about the potential use of antimicrobial films. Pathogen specificity may improve the antimicrobial efficacy while protecting microbes necessary for human health such as probiotic microbes and also bacteria controlling the growth of pathogenic bacteria [307]. Developing novel antimicrobial packaging materials with high specificity for targeting only pathogenic organisms and not affecting symbiotic bacteria was accomplished by Vonasek et al. [304]. Other antimicrobial agents such as essential oils, nisin, and the bioactive proteins lactoperoxidase, lactoferrin, and lysozyme have also been investigated [308,309,310,311]. Concerns about the incorporation of antimicrobial additives were pointed out by Chen [312] and Hotchkiss [313] who indicated potential negative effects on the film’s mechanical and optical properties. Ozdemir and Floros [314,315] have been working on optimizing the mechanical and optical properties of films containing preservatives with favorable results. Also, micro-encapsulated food additives in whey protein-based films were reported by Young, Sarda, and Rosenberg [316,317].

For ensuring high quality products the fabrication of films and coatings with antimicrobial properties is a suitable approach [306]. Gonzalez and Igarzabal [318] incorporated an antifungal agent and an antibacterial agent (natamycin and thymol respectively) into bilayer films produced from SPI and poly lactic acid (PLA). The SPI/PLA films had high transparency, strong adhesion between layers, suitable mechanical properties with respect to those of pure SPI films, and decreased WVP. The antibacterial additives were able to markedly inhibit the growth of mold, yeast, and two strains of bacteria. In another example, oregano or thyme essential oils reduced the counts of coliform and *Pseudomonas* spp*.* on beef patties [319]. Furthermore, soy protein films with incorporated grape seed extract exhibited inhibitory activity against *L. monocytogenes* [320]. With reference to other potential additives, the anti-oxidative effect of ferulic acid in soy protein films and coatings was reported by Ou et al. [321]. Further, Friesen et al. [322] recently reported that rutin and epicatechin can be used as crosslinking agents as a natural means for improving specific properties of SPI films. The addition of rutin increased the puncture strength, whereas epicatechin was found to increase the water vapor permeability.

#### 5.2.3. Addition of Lipid Materials

Due to the high water vapor permeability of whey protein films, much research has focused on improving the barrier properties of the films by the addition of hydrophobic materials such as waxes and lipids [310,323]. Shellhammer et al. [324] investigated carnauba wax, candelia wax, milkfat fraction, and beeswax (BW, a solid and highly hydrophobic lipid). When incorporated into whey protein films, the viscoelastic milk fat and beeswax improved the water vapor permeability more than candelia or carnauba wax. Perez-Gago and Krochta [325] found that the tensile strength and elongation significantly increased when the particle size of the beeswax in whey protein films decreased. Anker et al. [326] reported a 70-fold reduction of the water vapor permeability when acetylated monoglyceride was added to whey protein films. However, lipids were shown to also negatively affect the mechanical properties of other proteins. The tensile strength and elongation can decrease at high concentrations. Furthermore, the films can become brittle and hard to handle without breaking [324] and other features such as the transparency can be affected [327]. Edible whey protein films prepared with almond and walnut oils have been reported to have increased film opacity, even though they had improved water vapor barrier properties [328]. As numerous studies on the modification of the whey protein film structure by addition of waxes have indicated, lower lipid and wax contents and smaller particle sizes result in significantly enhanced WPI films [325,329,330,331]. Nevertheless, compared to synthetic films and coatings, WPI films still only have moderate moisture barriers even with the inclusion of lipids. With regard to potential applications, whey protein films may be best for foods that need a low to moderate moisture barrier [310]. Furthermore, WPI-based films containing oregano and pimento essential oils were reported to have anti-oxidative activity for meat [332]. Other anti-oxidative compounds were reported to be vitamin E for peanuts [333,334] and ascorbic acid for apples [335].

Early studies of Gontard et al. [336] have shown that the WVP of WG films can be optimized by incorporation of lipid materials (emulsion or bilayer film). The WVP was reduced by 200-fold compared to an uncoated control film as a result of beeswax being laminated onto the WG films. Gennadios, Weller, and Testin [337] added nonpolar hydrophobic substances to the film forming solution as well. Mineral oil was able to reduce the WVP by about 25%. However, the TS also decreased. In addition, wheat gluten coatings were improved by the incorporation of lipids (beeswax, stearic and palmitic acids) by Tanada-Palmu and Grosso [338], resulting in good decay-control properties for fruit. Lastly, increased hydrophobicity was also achieved through the addition of epoxidized soybean oil (ESO) under alkaline conditions [339].

#### 5.2.4. Other Bioactive Compounds

In 1998, Rhim et al. investigated the effect of dialdehyde starch (DAS) incorporated in SPI films. Due to water absorption by hydrophilic groups along the DAS polymer chains, small increases in the WVP were observed. However, with increased tensile strength and substantially reduced solubility in water, DAS showed the potential for increasing the resistance of SPI films, thus improving their functionality [340]. In order to improve the water barrier, SPI-based films have been combined with hydrophobic lipid materials. One approach is to add lipids to protein film forming solutions, which are then cast to prepare emulsified, bi-component or multi-component films [341]. Alternatively, molten lipids can be laminated onto protein films, resulting in bi-layer or multi-layer films [342]. Gennadios et al. [343] prepared soy protein-fatty acid bi-component films. The SPI films had notably lower WVP values than the control films. Unfortunately, the addition of fatty acids substantially reduced the film TS. Lipid oxidation may be another obstacle. This might be the reason why there is little literature on SPI-lipid composite films.

There have been many more attempts to fabricate composite SPI films and coatings, as elaborately reviewed by Koshy et al. [275]. A distinction was made between organic filler biocomposites (chitin, starch, and other polymers), organic filler bio-nanocomposites, and inorganic bio-nanocomposites. Readers are encouraged to refer to the relevant publication for further information about composite SPI films.

In another approach, Cao et al. [344] prepared composite films from SPI and gelatin and reported improved mechanical properties. The tensile strength, elongation at break, and elastic modulus were increased. Moreover, the films became more transparent and easier to handle. Guerrero et al. [53] incorporated gelatin into SPI-based films and reported similar results. The films showed higher tensile strength and similar elongation at break compared to the control. Moreover, the hydrophilicity decreased, while the UV barrier properties were maintained, suggesting a potential use of SPI films for the retardation of product oxidation induced by UV light.

An interesting approach was the addition of cysteine and gluten to soy protein films. Due to an increase in disulfide bond formation, the cysteine increased the tensile strength of soy:gluten (4:1) films. Furthermore, as gluten has large numbers of non-polar amino acids which contribute to hydrophobic interactions, its addition lowered the WVP [345].

## 6. Summary Tables

The properties of protein-based films are summarized in this section.

### 6.1. Mechanical Properties of Protein-Based Films

Table 3 lists the mechanical characteristics of different protein-based films. Readers are encouraged to refer to the respective publications for further information. See the bottom of the tables for descriptions of the footnotes.

### 6.2. Barrier Properties of Protein-Based Films

Table 4 gives an overview of the WVP and OP properties of different protein-based films. Readers are encouraged to refer to the respective publications for further information. See the bottom of the tables for descriptions of the footnotes.

## 7. Conclusions and Future Trends

All the physical, chemical, and biochemical methods that have been discussed have an effect on the films and coatings produced from the three protein sources. The resulting mechanical and barrier properties can therefore be adversely affected. However, the efficiency of the various techniques not only depends on their intensities but is also strongly influenced by the concentration and presence of other components and on the preparation methods.

Unfortunately, the respective methods often yield ambivalent results, meaning no single film or coating is appropriate for all applications for food protection. Different foods place different requirements on packaging materials. For example, the shelf life of fresh fruit or vegetables depends on the exchange of water vapor and other gas transport. On the other hand, products containing lipids, such as milk, have to be protected against oxidation by light and oxygen.

Even though there are some safety concerns about nanomaterials, nanotechnological research has recently been a most exciting development. Developments here are likely to elevate food packaging to new heights and capture the market within the next few years. Furthermore, in order to fully protect and extend the shelf life of a food product, the use of multiple components is, and will continue to be, imperative. The future challenge will therefore be to take advantage of the various technologies to form optimal protein-based films and coatings for each individual application.

With regards to the information collated for this paper, it can be concluded that the combination of nanotechnological advancements with enzymatic and physical treatments has the greatest promise.

However, it is possible to enhance the characteristics of protein-based films to such a level that they represent an interesting alternative to conventional petroleum-based films and coatings [280,347,348,349]. The general aim must remain biopolymer production on a large technical scale for industrial applications. Nevertheless, the use of protein based films and coatings is not limited to packaging material. Recent studies and reviews on protein-based adhesives [350,351,352,353,354,355,356,357,358], protectants for building materials [358], thermoplastic composites [359], bioelectronics [360], heat sealable [361] and microorganism carrier films [362] show some future prospects of these upcoming biopolymer materials.

## Figures and Tables

**Table 1:** Prolamin and glutelin proteins of whey gluten divided into high, medium, and low molecular mass groups with their subunits and their wheat gluten and cysteine contents [62].

Group	High Molecular		Medium Molecular		Low Molecular		
	HMW-Subunits		ω-Gliadin		Gliadin		LMW-Subunit
	x-type	y-type	ω5	ω1,2	α	γ	
Gluten protein content (%)	4–9	3–4	3–6	4–7	28–33	23–31	19–25
Sum of cysteine	4	7	0	0	6	8	8

**Table 2:** Amino acid composition and commonly used modifications [207,208].

Side Chain	Amino Acid	Commonly Used Modifications	β-Lactoglobulin (Whey Protein) (mol %)	β-Conglycinin (Soy Protein) (mol %)	γ-Gliadins (Wheat Gluten) (mol %)
Amino	Lysine	Alkylation, Acylation, TG ^1^	10.5	6.1	-
	Arginine		2.5	8.3	1.8
Carboxyl	Glutamine	Amidation, Esterification	11.2	-	-
	Glutamic acid	TG ^1^	6.2	24.5	45.8
	Asparagine		3.1	12.0	2.9
	Aspartic acid		6.9	-	-
Disulfide	Cysteine	Reduction, Oxidation	2.8	0.03	-
Imiazole	Histidine	Alkylation, Oxidation	1.5	2.8	1.6
Indole	Tryptophan	Alkylation, Oxidation	2.0	-	-
Phenolic	Tyrosine	Acylation, electrophilic	3.6	3.5	3.5
	Tryptophan	Substitution	2.0	-	-
	Histidine	-	1.5	2.8	1.6
	Phenylalanine	-	3.2	5.4	5.2
Sulfhydryl	Cysteine	Alkylation, Oxidation	2.8	0.03	-
Thioether	Methionine	Alkylation, Oxidation	-	-	-

^1^ Transglutaminase crosslinking: Isopeptide bonding between glutamine and lysine.

**Table 3:** Mechanical properties of protein-based films.

Film Modification	Tensile Strength (MPa)	Elongation (%)	Elastic Modulus (MPa)	Source
**Heating**				
**Heat treatment of solution**				
WPI: 70 °C:5 min	3.4 ± 0.4	7 ± 1	156 ± 17	[79]
WPI: 70 °C:10 min	3.3 ± 0.1	8 ± 2	141 ± 10	[79]
WPI: 70 °C:15 min	4.5 ± 0.2	9 ± 0.4	194 ± 12	[79]
WPI: 70 °C:20 min	4.9 ± 0.2	17 ± 5	192 ± 13	[79]
WPI: 80 °C:5 min	3.8 ± 0.3	7 ± 1	159 ± 24	[79]
WPI: 80 °C:10 min	7 ± 2	18 ± 3	299 ± 62	[79]
WPI: 80 °C:15 min	12 ± 2	17 ± 4	346 ± 71	[79]
WPI: 80 °C:20 min	14 ± 2	18 ± 4	460 ± 42	[79]
WPI: 90 °C:5 min	8 ± 2	3 ± 1	327 ± 71	[77]
WPI: 90 °C:10 min	12 ± 2	14 ± 3	429 ± 59	[79]
WPI: 90 °C:15 min	12 ± 2	16 ± 3	427 ± 41	[79]
WPI: 90 °C:20 min	13 ± 2	16 ± 5	472 ± 57	[79]
WPI: 90 °C:30 min	6.9	41	199	[75]
WPI: 100 °C:5 min	8 ± 3	14 ± 3	342 ± 32	[79]
WPI: 100 °C:10 min	10 ± 3	14 ± 4	419 ± 53	[79]
WPI: 100 °C:15 min	12 ± 2	15 ± 5	425 ± 28	[79]
WPI: 100 °C:20 min	9 ± 2	18 ± 3	429 ± 28	[79]
SPI control	11.2 ± 2.0	10.2 ± 5.5	928 ± 233	[84]
SPI: 85 °C	12.8 ± 2.7	16.8 ± 6.6	992 ± 276	[84]
**Heat treatment of the film**				
SPI: 60 °C:24 h	11 ^a^	180 ^a^	n/a	[23]
SPI: 70 °C:24 h	9 ^a^	170 ^a^	n/a	[23]
SPI: 80 °C:24 h	13 ^a^	160 ^a^	n/a	[23]
SPI control	8.2 ± 0.2	30 ± 3.3	n/a	[78]
SPI: 90 °C:24 h	14.7 ± 0.4	6.1 ± 0.7	n/a	[78]
WG control	1.7 ± 0.3	501 ± 46	13 ± 3	[27]
WG: 80 °C:15 min	2.4 ± 0.4	391 ± 58	29 ± 13	[27]
WG: 95 °C:15 min	2.5 ± 0.3	386 ± 75	30 ± 11	[27]
WG: 110 °C:15 min	3.1 ± 0.4	327 ± 58	37 ± 10	[27]
WG: 125 °C:15 min	6.3 ± 0.5	275 ± 12	40 ± 14	[27]
WG: 140 °C:15 min	7.3 ± 1.2	170 ± 26	98 ± 16	[27]
WG: 140 °C:1.5 min	4.2 ± 1.1	326 ± 49	37 ± 8	[27]
**Ultrasound**				
WPC control	3.36 ± 0.24	n/a	n/a	[151]
WPC: 168 kHz:1.9 W:0.5 h	2.75 ±0.32	n/a	n/a	[151]
WPC: 168 kHz:1.9 W:1 h	4.40 ± 0.20	n/a	n/a	[151]
WPC: 520 kHz:3 W:0.5 h	3.67 ± 0.47	n/a	n/a	[151]
WPC: 520 kHz:3 W:1 h	4.92 ± 0.72	n/a	n/a	[151]
WPC: 168 kHz:3.35 W:0.5 h	1.75 ± 0.85	n/a	n/a	[151]
WPC: 168 kHz:3.35 W:1 h	3.08 ± 0.28	n/a	n/a	[151]
WPC: 520 kHz:5.22 W:0.5 h	2.75 ± 0.36	n/a	n/a	[151]
WPC: 520 kHz:5.22 W:0.5 h	4.47 ± 0.14	n/a	n/a	[151]
WPI control	1.1 ^a^	n/a	19 ^a^	[150]
WPI: amplitude 16 µm	1.1 ^a^	n/a	18 ^a^	[150]
WPI: amplitude 80 µm	1.1 ^a^	n/a	20 ^a^	[150]
WPI: amplitude 160 µm	1.2 ^a^	n/a	17 ^a^	[150]
**UV and γ-irradiation**				
**UV irradiation of solution**				
WPI control	4.68 ± 0.39	114.0 ± 14.2	n/a	[173]
WPI: 324 J·cm^−2^	6.40 ± 0.30	110.0 ± 15.5	n/a	[173]
SPI control	n/a	n/a	299 ± 13	[174]
SPI: 125 W:2 h	n/a	n/a	309 ± 27	[174]
**UV irradiation of the film**				
SPI control	3.7 ^a^	124.2 ^a^	n/a	[162]
SPI: 51.8 J·m^−2^	5.25 ^a^	113 ^a^	n/a	[162]
WPI control	6.8 ^MD^	42 ^MD^	118 ^MD^	[13]
WPI: 2.3 J·cm^−2^	8.2 ^MD^	50 ^MD^	125 ^MD^	[13]
WPI: 10.2 J·cm^−2^	8.1 ^MD^	53 ^MD^	95 ^MD^	[13]
WPI: 19.0 J·cm^−2^	8 ^MD^	54 ^MD^	62 ^MD^	[13]
WPI: 31.4 J·cm^−2^	8.8 ^MD^	39 ^MD^	115 ^MD^	[13]
SPI control	n/a	n/a	299 ± 13	[174]
SPI: 125 W:2 h	n/a	n/a	256 ± 42	[174]
SPI control	8.2 ± 0.2	30 ± 3.3	n/a	[78]
SPI: 51.8 J·m^−2^	10.0 ± 0.6	23.3 ± 5.6	n/a	[78]
WG control	1.2 ± 0.2	n/a	n/a	[163]
WG: 51.8 J·m^−2^	2.0 ± 0.1	n/a	n/a	[163]
WG control	1.7 ± 0.3	501 ± 46	13 ± 3	[27]
WG: 0.25 J·cm^−2^	2.0 ± 0.3	424 ± 97	18 ± 6	[27]
WG: 1 J·cm^−2^	2.0 ± 0.3	478 ± 70	15 ± 4	[27]
**γ-irradiation of the film**				
WG control	2.1 ± 0.5	384 ± 82	29 ± 4	[27]
WG: 10 kGy	3.0 ± 0.7	261 ± 82	52 ± 15	[27]
WG: 20 kGy	2.6 ± 0.4	344 ± 45	36 ± 6	[27]
WG: 40 kGy	2.7 ± 0.4	297 ± 54	38 ± 7	[27]
**Compression Molding**				
WPI: 30% Gly:0.81 MPa:113 °C	7.5 ^a^	35 ^a^	180 ^a^	[82]
WPI: 30% Gly:0.81 MPa:127 °C	10.5 ^a^	35 ^a^	275 ^a^	[82]
WPI: 30% Gly:0.81 MPa:140 °C	10 ^a^	38 ^a^	245 ^a^	[82]
WPI: 30% Gly:2.25MPa:113 °C	5.5 ^a^	57 ^a^	125 ^a^	[82]
WPI: 30% Gly:2.25 MPa:127 °C	10 ^a^	50 ^a^	250 ^a^	[82]
WPI: 30% Gly:2.25 MPa:140 °C	10.5 ^a^	48 ^a^	250 ^a^	[82]
**Chemical Modifications**				
**Acylation**				
SPI control	2.5	n/a	n/a	[218]
SPI:acetylated	2.5	n/a	n/a	[218]
SPI:succinylated	2.6	n/a	n/a	[218]
**Alkylation**				
SPI:PGA:pH 8	0.779 ± 0.076	18.3 ± 0.9	n/a	[209]
SPI:PGA:KOH	0.848 ± 0.069	22.8 ± 1.5	n/a	[209]
**Hydrolysis**				
WPI control	4.35 ^a^	75.8 ^a^	140 ^a^	[185]
WPI:5.5% DH	0.5 ^a^	31.5 ^a^	3 ^a^	[185]
WPI:10% DH	1.5 ^a^	7.7 ^a^	50 ^a^	[185]
**pH alteration**				
WPI:pH 7	n/a	40 ± 4	78 ± 3	[236]
WPI:pH 8	n/a	54 ± 5	74 ± 2	[236]
WPI:pH 9	n/a	66 ± 7	64 ± 2	[236]
SPI:pH 6	3.5 ± 0.2	72 ± 13.2	n/a	[232]
SPI:pH 8	3.6 ± 0.4	139.5 ± 19.5	n/a	[232]
SPI:pH 10	3.6 ± 0.1	169.3 ± 9.3	n/a	[232]
SPI:pH 12	1.3 ± 0.5	66.5 ± 31.6	n/a	[232]
**Enzymatic Crosslinking**				
WPI control	5.64 ± 0.64	n/a	n/a	[251]
WPI:TG	12.53 ± 1.12	n/a	n/a	[251]
WPI:SPI 11S control	6.26 ± 0.88	n/a	n/a	[251]
WPI:SPI 11S:TG	17.86 ± 1.44	n/a	n/a	[251]
WPI control	4 ^a^	25 ^a^	n/a	[261]
WPI:TG	3.2 ^a^	105 ^a^	n/a	[261]
WPI:Chitosan 0.25 mg·cm^−2^	9.5 ± 0.6	14.1 ± 0.59	n/a	[262]
WPI:Chitosan:TG	26.2 ± 0.9	3.1 ± 0.3	n/a	[262]
SPI 11S control	7.61 ± 0.71	n/a	n/a	[251]
SPI 11S:TG	16.4 ± 1.38	n/a	n/a	[251]
SPI control	11.2 ± 2.0	10.2 ± 5.5	928 ± 233	[84]
SPI:Horseradish Peroxidase	n/a	1.1 ± 0.5	1503 ± 129	[84]
SPI:Pectin control	6.8 ± 0.92	11.61 ± 1.09	n/a	[270]
SPI:Pectin:TG	12.4 ± 1.05	7.2 ± 1.03	n/a	[270]
SPI:Gly control	2.21 ± 0.25	159.87 ± 9.20	n/a	[257]
SPI:Gly:mTG	2.58 ± 0.28	105.88 ± 9.20	n/a	[257]
SPI:Gly:Sorbitol control	2.58 ± 0.11	102.04 ± 13.68	n/a	[257]
SPI:Gly:Sorbitol:mTG	3.10 ± 0.17	80.04 ± 5.40	n/a	[257]
SPI:Sorbitol control	4.16 ± 0.04	101.77 ± 15.60	n/a	[257]
SPI:Sorbitol:mTG	4.48 ± 0.35	27.33 ± 3.61	n/a	[257]
SPI:mTG 0 U·g^−1^	3.12 ± 0.25	167.1 ± 22.8	n/a	[271]
SPI:mTG 4 U·g^−1^	3.75 ± 0.30	124.4 ± 11.8	n/a	[271]
SPI:mTG 10 U·g^−1^	3.98 ± 0.20	108.1 ± 9.1	n/a	[271]
SPI:mTG 40 U·g^−1^	1.75 ± 0.27	83.3 ± 12.1	n/a	[271]
SPI:mTG 60 U·g^−1^	0.95 ± 0.18	57.4 ± 7.8	n/a	[271]
SPI:Gelatin control	33.78 ± 5.81	39.17 ± 6.76	n/a	[274]
SPI:Gelatin:TG 10%	65.73 ± 7.07	32.93 ± 7.04	n/a	[274]
SPI:Gelatin:TG 20%	45.32 ± 5.79	14.20 ± 3.66	n/a	[274]
SPI:Gelatin:TG 30%	42.74 ± 4.15	12.12 ± 4.29	n/a	[274]
WG:Putrescine control	1.12 ± 0.11	327 ± 32	6.76 ± 2.99	[276]
WG:Putrescine 0.09 mol:mol TG	1.61 ± 0.16	582 ± 57	2.15 ± 0.62	[276]
WG:Cadaverine control	0.94 ± 0.06	304 ± 73	9.51 ± 3.40	[276]
WG:Cadaverine 0.09 mol:mol TG	1.24 ± 0.15	522 ± 83	2.04 ± 0.34	[276]
WG:Diaminohexane control	0.89 ± 0.06	351 ± 81	6.50 ± 1.68	[276]
WG:Diaminohexane 0.09 mol:mol TG	1.87 ± 0.25	527 ± 85	3.96 ± 0.91	[276]
WG:Diaminooctance control	0.75 ± 0.06	413 ± 118	4.18 ± 1.43	[276]
WG:Diaminohectane 0.09 mol:mol TG	1.53 ± 0.24	501 ± 73	2.92 ± 0.15	[276]
**Composite Films and Bioactive Compounds**				
WPI control	28.0 ^a^	1.8 ^a^	1675 ^a^	[324]
WPI:40% carnauba wax	22.5 ^a^	1.5 ^a^	1700 ^a^	[324]
WPI:40% candelilla wax	17.0 ^a^	1.0 ^a^	1800 ^a^	[324]
WPI:40% milk fat fraction	19.0 ^a^	2.2 ^a^	975 ^a^	[324]
WPI:40% BW	18.5 ^a^	2.0 ^a^	1200 ^a^	[324]
WPI:Gly:BW (60:20:20) PS 0.5 µm	10.2 ± 0.8 ^a^	4.9 ± 1.3 ^a^	550 ± 20 ^a^	[325]
WPI:Gly:BW (30:10:60) PS 0.5 µm	5.5 ± 0.2 ^a^	3 ± 0.2 ^a^	430 ± 30 ^a^	[325]
WPI:Gly:BW (30:10:60) PS 1.0 µm	4.2 ± 0.3 ^a^	2.7 ± 0.3 ^a^	420 ± 10 ^a^	[325]
WPI:Gly:BW (30:10:60) PS 1.5 µm	3.0 ± 0.2 ^a^	1.2 ± 0.2 ^a^	410 ± 30 ^a^	[325]
WPI:Gly:BW (30:10:60) PS 2.0 µm	2.9 ± 0.05 ^a^	1.2 ± 0.2 ^a^	380 ± 15 ^a^	[325]
WPI control	2.2 ± 0.11	20 ± 3	96 ± 3.7	[326]
WPI:Acetem	1.0 ± 0.08	29 ± 5	36 ± 3.3	[326]
WPI control	7.1 ± 1.8	29.8 ± 6.5	1.9 ± 0.9	[328]
WPI:Almond oil 0.5%	10.2 ± 1.9	21.9 ± 2.6	3.6 ± 0.7	[328]
WPI:Almond oil 1.0%	5.4 ± 0.8	53.7 ± 7.7	1.4 ± 0.2	[328]
WPI:Walnut oil 0.5%	11.8 ± 0.9	14.5 ± 4.5	3.8 ± 0.6	[328]
WPI:Walnut oil 1.0%	6.9 ± 1.5	24.9 ± 4.9	2.3 ± 0.6	[328]
WPI:Gly:(1:1)	2.9 ± 0.4	118 ± 20 ^a^	41 ± 4	[329]
WPI:BW:Gly (1:1:1)	1.2 ± 0.1	30 ± 10 ^a^	39 ± 12	[329]
WPI:CW:Gly (1:1:1)	3.1 ± 0.1	10 ± 1 ^a^	124 ± 29	[329]
WPI control	2.0 ± 0.1 ^a^	60 ± 5 ^a^	48 ± 3 ^a^	[331]
WPI:Gly:CAN 7.5 g:100 g	0.6 ± 0.1 ^a^	65 ± 5 ^a^	45 ± 3 ^a^	[331]
WPI:Gly extruded	2.4 ± 0.1 ^a^	90 ± 5 ^a^	30 ± 1 ^a^	[331]
WPI:Gly:CAN 7.5 g:100 g extruded	1.7 ± 0.2 ^a^	55 ± 5 ^a^	20 ± 1 ^a^	[331]
WPI:Gly compressed extruded	2.1 ± 0.1 ^a^	60 ± 5 ^a^	15 ± 1 ^a^	[331]
WPI:Gly:CAN 7.5 g:100 g compressed extruded	1.5 ± 0.1 ^a^	30 ± 5 ^a^	10 ± 2 ^a^	[331]
WPI:TiO_2_ 0% *w*/*w*	6 ± 1.5 ^a^	13 ± 1 ^a^	n/a	[281]
WPI:TiO_2_ 1% *w*/*w*	8.25 ± 0.25 ^a^	10 ± 2 ^a^	n/a	[281]
WPI:TiO_2_ 0 wt %	1.69 ± 0.03	55.56 ± 1.05	31.44 ± 3.03	[283]
WPI:TiO_2_ 1 wt %	2.19 ± 3.03	40.11 ± 1.01	63.09 ± 1.98	[283]
WPI:TiO_2_ 4 wt %	1.78 ± 0.08	12.14 ± 0.22	39.23 ± 3.65	[283]
WPI control	3.40 ± 0.58	50.9 ± 12.5	171.8 ± 14.3	[284]
WPI:Cloisite Na^+^	2.98 ± 0.29	42.4 ± 7.6	109.3 ± 18.0	[284]
WPI:Cloisite 30B	3.29 ± 0.10	51.7 ± 4.8	162.6 ± 37.9	[284]
WPI:Cloisite 20A	1.55 ± 0.32	29.1 ± 9.0	115.5 ± 13.5	[284]
WPI:ZNP 0 *w*/*w*	2.5 ± 0.1	50 ± 10 ^a^	n/a	[285]
WPI:ZNP 0.4 *w*/*w*	3.9 ± 0.1 ^a^	50 ± 5 ^a^	n/a	[285]
WPI:ZNP 1.2 *w*/*w*	10.2 ± 0.3	35 ± 5 ^a^	n/a	[285]
WPI control	1.902 ± 0.123	126.8 ± 3.9	20.21 ± 2.26	[308]
WPI:lactoperoxidase system 0.7% *w*/*w*	2.090 ± 0.156	129.5 ± 3.4	22.57 ± 2.57	[308]
WPI:Sorbitol (50:50)	3.32	27.60	83.96	[314]
WPI:Sorbitol:BW (50:35:15)	3.91	12.5	129.72	[314]
WPI:Sorbitol:Potassium sorbate (50:43.3:6.7)	2.61	68.9	28.47	[314]
WPI:Sorbitol:BW:Potassium sorbate (50:35:11.7:3.3)	3.86	32.8	90.27	[314]
SPI:WG (2:1):pH 7.0	4.85 ± 0.20	n/a	n/a	[345]
SPI:WG (2:1): cys:pH 7.0	4.85 ± 0.20	n/a	n/a	[345]
SPI:WG (3:1):pH 7.0	5.68 ± 0.20	n/a	n/a	[345]
SPI:WG (3:1):cys:pH 7.0	6.37 ± 0.20	n/a	n/a	[345]
SPI:WG (4:1):pH 7.0	5.04 ± 0.20	n/a	n/a	[345]
SPI:WG (4:1):cys:pH 7.0	6.87 ± 0.20	n/a	n/a	[345]
SPI:WG (1:0) :pH 7.0	3.70 ± 0.20	n/a	n/a	[345]
SPI:WG (1:0):cys:pH 7.0	6.72 ± 0.22	n/a	n/a	[345]
SPI: DAS 0%	6.34 ± 0.02	65.9 ± 25.3	n/a	[340]
SPI: DAS 10%	7.84 ± 0.19	59.8 ± 19.9	n/a	[340]
SPI:10% fatty acid	4.4 ± 0.6	70.1 ± 9.8	n/a	[343]
SPI:10% lauric acid	1.9 ± 0.1	125.8 ± 25.0	n/a	[343]
SPI:10% myristic acid	0.4 ± 0.1	41.0 ± 5.4	n/a	[343]
SPI:10% palmitic acid	1.2 ± 0.1	39.0 ± 15.3	n/a	[343]
SPI:10% oleic acid	2.2 ± 0.2	228.1 ± 14.2	n/a	[343]
SPI:Gelatin (10:0)	5 ^a^	2.30 ± 1.0 ^a^	750 ^a^	[344]
SPI:Gelatin (8:2)	25.55	2.64 ± 0.4	1280	[344]
SPI:Gelatin (6:4)	30.00 ^a^	3.1 ± 0.4 ^a^	1500.00 ^a^	[344]
SPI:Gelatin (4:6)	35.00 ^a^	3.1 ± 0.8 ^a^	1550.00 ^a^	[344]
SPI:Gelatin (2:8)	44.6	3.23	1861.16	[344]
SPI:Gelatin (0:10)	75 ^a^	3.50 ± 0.1 ^a^	3000 ^a^	[344]
SPI:BW:Span	90.8	25.8	n/a	[341]
WG control	2.6 ± 0.2	237.9 ± 21.9	n/a	[337]
WG:Mineral oil	2.2 ± 0.3	267.2 ± 40.1	n/a	[337]
WG:MMT 0 wt %	1.86 ± 0.48	58.4 ± 7.0	3.73 ± 0.48	[300]
WG:MMT 2.5 wt %	2.37 ± 0.44	55.4 ± 15.4	5.58 ± 1.04	[300]
WG:MMT 5.0 wt %	4.70 ± 0.84	16.0 ± 12.4	10.58 ± 3.43	[300]
WG:MMT 7.5 wt %	3.60 ± 0.42	15.2 ± 2.3	11.44 ± 1.77	[300]
WG:MMT 10.0 wt %	n/a	n/a	n/a	[300]
WG control	n/a	n/a	n/a	[301]
WG:MMT 2.5% C0	n/a	n/a	n/a	[301]
WG:MMT 5.0% C0	n/a	n/a	n/a	[301]
WG:HEC 0%	1.20 ± 0.1 ^a^	210 ± 15 ^a^	10 ^a^	[346]
WG:HEC 5%	1.10 ± 0.05 ^a^	160 ± 20 ^a^	10 ^a^	[346]
WG:HEC 15%	1.10 ± 0.05 ^a^	120 ± 10 ^a^	13 ^a^	[346]
WG:HEC 31.8%	2.4 ± 0.05 ^a^	45 ± 5 ^a^	65 ^a^	[346]
**Nanotechnology**				
SPI control	1.10 ± 0.20	65.95 ± 17.76	26.89 ± 11.21	[290]
SPI:SNC 5%	1.34 ± 0.07	58.67 ± 9.88	39.42 ± 9.93	[290]
SPI:SNC 20%	2.61 ± 0.26	41.89 ± 8.61	102.23 ± 14.93	[290]
SPI:SNC 40%	5.08 ± 0.48	21.35 ± 10.54	310.34 ± 21.55	[290]
SPI:Cloisite 20A 0%	2.26 ± 0.48	11.85 ± 0.39	n/a	[295]
SPI:Cloisite 20A 5%	12.40 ± 0.65	42.80 ± 0.57	n/a	[295]
SPI:Cloisite 20A 10%	14.15 ± 0.33	71.00 ± 3.68	n/a	[295]
SPI:Cloisite 20A 15%	13.66 ± 0.28	22.80 ± 1.70	n/a	[295]
SPI:Cloisite 30B 0%	2.26 ± 0.48	11.85 ± 0.39	n/a	[295]
SPI:Cloisite 30B 5%	15.11 ± 0.86	81.60 ± 2.83	n/a	[295]
SPI:Cloisite 30B 10%	16.19 ± 0.75	103.60 ± 4.53	n/a	[295]
SPI:Cloisite 30B 15%	18.64 ± 0.23	54.80 ± 2.26	n/a	[295]
SPI control	2.26 ± 0.48	11.85 ± 0.39	n/a	[295]
SPI:MMT 5%	6.28 ± 0.88	64.60 ± 4.69	n/a	[295]
SPI:MMT 10%	12.62 ± 0.54	23.98 ± 5.02	n/a	[295]
SPI:MMT 15%	15.60 ± 1.69	17.80 ± 2.27	n/a	[295]
SPI:MMT 0 wt %	8.5 ^a^	90 ^a^	180 ^a^	[297]
SPI:MMT 4 wt %	10.5 ^a^	65 ^a^	260 ^a^	[297]
SPI:MMT 8 wt %	12 ^a^	30 ^a^	330 ^a^	[297]
SPI:MMT 12 wt %	14 ^a^	17 ^a^	410 ^a^	[297]
SPI:MMT 16 wt %	15 ^a^	8 ^a^	520 ^a^	[297]
SPI:MMT 20 wt %	14.5 ^a^	5 ^a^	590 ^a^	[297]
SPI:MMT 0%	3.0 ^a^	37.5 ^a^	42 ^a^	[298]
SPI:MMT 2.5%	4.5 ^a^	32.5 ^a^	n/a	[298]
SPI:MMT 5%	5.5 ^a^	27.5 ^a^	n/a	[298]
SPI:MMT 7.5%	8 ^a^	20.0 ^a^	n/a	[298]
SPI:MMT 10%	8.5 ^a^	6.5 ^a^	n/a	[298]
SPI control	1.35 ^a^	60 ^a^	n/a	[299]
SPI:PNP 0.5%	2.00 ^a^	85 ^a^	n/a	[299]
SPI:PNP 1.0%	2.10 ^a^	86 ^a^	n/a	[299]
SPI:PNP 2.0%	3.55 ^a^	90 ^a^	n/a	[299]
SPI:PNP 4.0%	2.70 ^a^	82 ^a^	n/a	[299]
**Antimicrobial Films**				
SPI control	1.08 ± 0.34	24.63 ± 0.13	22.80 ± 6.14	[318]
SPI:PLA (60:40)	8.57 ± 1.61	1.09 ± 0.09	1085 ± 134	[318]
SPI:PLA (50:50)	13.69 ± 0.94	1.25 ± 0.02	1579 ± 52	[318]
SPI control	9.3	115.7	n/a	[322]
SPI:Rutin	35.1	73.5	n/a	[322]
SPI:Epicatechin	22.1	38.5	n/a	[322]

Some data are converted to the same units; ^a^ Numerical value estimated from graph; ^MD^ Measured in Machine Direction; n/a not available.

**Table 4:** Barrier properties of protein-based films.

Film Modification	Water Vapor Permeability (g·m·m^−2^·s^−1^ Pa^−1^)	Relative Humidity (%)	Oxygen Permeability (cm^3^·m^−2^·d^−1^·bar^−1^)	Source
**Heating**				
**Heat treatment of solution**				
WPI control	1.41 × 10^−9^ ^a^	n/a	n/a	[75]
WPI: 90 °C:30 min	1.38 × 10^−9^ ^a^	n/a	n/a	[75]
WPI control	n/a	n/a	(6.83 ± 0.35) × 10^−13^	[79]
WPI: 90 °C:30 min	n/a	n/a	(9.03 ± 0.35) × 10^−13^	[79]
SPI control	5.06 × 10^−10^	n/a	n/a	[84]
SPI: 85 °C	4.48 × 10^−10^	n/a	n/a	[84]
**Heat treatment of the film**				
SPI: 60 °C:24 h	2.3 × 10^−9^ ^a^	0–50	n/a	[23]
SPI: 70 °C:24 h	2.3 × 10^−9^ ^a^	0–50	n/a	[23]
SPI: 80 °C:24 h	1.8 × 10^−9^ ^a^	0–50	n/a	[23]
WG control	(1.37 ± 0.18) × 10^−10^	0–100	n/a	[27]
WG: 80 °C:15 min	(1.22 ± 0.07) × 10^−10^	0–100	n/a	[27]
WG: 95 °C:15 min	(1.13 ± 0.13) × 10^−10^	0–100	n/a	[27]
WG: 110 °C:15 min	(1.19 ± 0.14) × 10^−10^	0–100	n/a	[27]
WG: 125 °C:15 min	(1.37 ± 0.07) × 10^−10^	0–100	n/a	[27]
WG: 140 °C:15 min	(1.15 ± 0.04) × 10^−10^	0–100	n/a	[27]
WG: 140 °C:1.5 min	(1.21 ± 0.23) × 10^−10^	0–100	n/a	[27]
**UV and γ-Irradiation**				
**UV Irradiation of Solution**				
WPI control	(4.49 ± 0.38) × 10^−10^	0–90	(1.04 ± 0.06) × 10^−7^	[173]
WPI: 324 J·cm^−2^	(4.98 ± 0.24) × 10^−10^	0–90	(9.17 ± 0.09) × 10^−8^	[173]
**UV Irradiation of the Film**				
WPI control	349 ± 9 ^b^	50–0	n/a	[13]
WPI: 2.3 J·cm^−2^	511 ± 11 ^b^	50–0	n/a	[13]
WPI: 10.2 J·cm^−2^	434 ± 23 ^b^	50–0	n/a	[13]
WPI: 19.0 J·cm^−2^	383 ± 40 ^b^	50–0	n/a	[13]
WPI: 31.4 J·cm^−2^	386 ± 19 ^b^	50–0	n/a	[13]
SPI control	(2.0 ± 0.2) × 10^−9^	50–100	n/a	[162]
SPI: 51.8 J·cm^−2^	(2.5 ± 0.8) × 10^−9^	50–100	n/a	[162]
WG control	(4.44 ± 0.14) × 10^−9^	50–100	n/a	[163]
WG: 51.8 J·cm^−2^	(4.19 ± 0.08) × 10^−9^	50–100	n/a	[163]
WG control	(1.37 ± 0.18) × 10^−10^	0–100	n/a	[27]
WG: 0.25 J·cm^−2^	(1.42 ± 0.09) × 10^−10^	0–100	n/a	[27]
WG: 1 J·cm^−2^	(1.35 ± 0.07) × 10^−10^	0–100	n/a	[27]
**γ-Irradiation of Solution**				
WPI control	3.5 × 10^−10^ ^a^	56–100	n/a	[177]
WPI: 32 kGy	2.6 × 10^−10^ ^a^	56–100	n/a	[177]
WPC control	3.0 × 10^−10^ ^a^	56–100	n/a	[177]
WPC: 32 kGy	1.7 × 10^−10^	56–100	n/a	[177]
**γ-Irradiation of the Film**				
WG control	(1.15 ± 0.02) × 10^−10^	0–100	n/a	[27]
WG: 10 kGy	(1.37 ± 0.04) × 10^−10^	0–100	n/a	[27]
WG: 20 kGy	(1.37 ± 0.09) × 10^−10^	0–100	n/a	[27]
WG: 40 kGy	(1.21 ± 0.07) × 10^−10^	0–100	n/a	[27]
**Chemical Modifications**				
**Acylation**				
SPI	0.84 × 10^−9^	100/60	0.7 × 10^−16^	[218]
SPI: acetylated	0.86 × 10^−9^	100/60	0.9 × 10^−16^	[218]
SPI: succinylated	0.84 × 10^−9^	100/60	0.7 × 10^−16^	[218]
**Hydrolysis**				
WPI control	1.29 × 10^−9^ ^a^	50 ± 1	1.2 × 10^−12^ ^a^	[185]
WPI: 5.5% DH	1.25 × 10^−9^ ^a^	50 ± 1	1.4 × 10^−12^ ^a^	[185]
WPI: 10% DH	1.35 × 10^−9^ ^a^	50 ± 1	1 × 10^−12^ ^a^	[185]
**pH Alteration**				
WPI:pH 4 (pI)	1.39 × 10^−9^ ^a^	n/a	n/a	[237]
WPI:pH 5 (pI)	1.89 × 10^−9^ ^a^	n/a	n/a	[237]
WPI:pH 6	1.36 × 10^−9^ ^a^	n/a	n/a	[237]
WPI:pH 7	1.25 × 10^−9^ ^a^	n/a	n/a	[237]
WPI:pH 8	1.36 × 10^−9^ ^a^	n/a	n/a	[237]
SPI:pH 6 (pI)	3.04 × 10^−9^	n/a	1.0 × 10^−16^	[232]
SPI:pH 8	1.90 × 10^−9^	n/a	0.59 × 10^−16^	[232]
SPI:pH 10	2.54 × 10^−9^	n/a	0.43 × 10^−16^	[232]
SPI:pH 12	1.79 × 10^−9^	n/a	0.37 × 10^−16^	[232]
**Enzymatic Crosslinking**				
WPI control	(7.53 ± 1.47) × 10^−10^	100–50	n/a	[251]
WPI:TG	(1.36 ± 0.17) × 10^−9^	100–50	n/a	[251]
WPI:SPI 11S control	(1.36 ± 0.15) × 10^−9^	100–50	n/a	[251]
WPI:SPI 11S:TG	(2.13 ± 0.17) × 10^−9^	100–50	n/a	[251]
WPI control	4.5 × 10^−12^ ^a^	n/a	n/a	[261]
WPI:TG	3.4 × 10^−12^ ^a^	n/a	n/a	[261]
WPI:Chitosan 0.25 mg·cm^−2^	3.24 ± 0.15	0	(2.38 ± 0.08) × 10^−10^	[262]
WPI:Chitosan:TG	0.88 ± 0.06	0	(9.03 ± 0.93) × 10^−11^	[262]
WPI control	n/a	50	2.11 × 10^−9^	[268]
WPI:TG 10 units·g^−1^	n/a	50	3.47 × 10^−10^	[268]
WPI control	(7.53 ± 1.47) × 10^−10^	100–50	n/a	[251]
WPI:TG	(1.36 ± 0.17) × 10^−9^	100–50	n/a	[251]
WPI:SPI 11S control	(1.36 ± 0.15) × 10^−9^	100–50	n/a	[251]
WPI:SPI 11S:TG	(2.13 ± 0.17) × 10^−9^	100–50	n/a	[251]
SPI 11S control	(1.33 ± 0.25) × 10^−9^	100–50	n/a	[251]
SPI 11S:TG	(2.17 ± 0.16) × 10^−9^	100–50	n/a	[251]
SPI control	5.06 × 10^−10^	n/a	n/a	[84]
SPI:Horseradish Peroxidase	n/a	n/a	n/a	[84]
SPI:Gly control	(3.44 ± 0.03) × 10^−10^	100–0	n/a	[257]
SPI:Gly:mTG	(3.69 ± 0.14) × 10^−10^	100–0	n/a	[257]
SPI:Gly:Sorbitol control	(2.94 ± 0.14) × 10^−10^	100–0	n/a	[257]
SPI:Gly:Sorbitol:mTG	(3.78 ± 0.03) × 10^−10^	100–0	n/a	[257]
SPI:Sorbitol control	(3.25 ± 0.14) × 10^−10^	100–0	n/a	[257]
SPI:Sorbitol:mTG	(3.64 ± 0.00) × 10^−10^	100–0	n/a	[257]
SPI:Gelatin control	(1.84 ± 0.03) × 10^−10^	100–0	n/a	[274]
SPI:Gelatin:TG 10%	(1.78 ± 0.03) × 10^−10^	100–0	n/a	[274]
SPI:Gelatin:TG 20%	(1.69 ± 0.04) × 10^−10^	100–0	n/a	[274]
SPI:Gelatin:TG 30%	(1.63 ± 0.03) × 10^−10^	100–0	n/a	[274]
**Composite Films and Bioactive Compounds**				
WPI control	5.21 × 10^−10^ ^a^	100–0	n/a	[324]
WPI: 40% carnauba wax	(3.82 ± 0.30) × 10^−10^	100–0	n/a	[324]
WPI: 40% candelilla wax	(3.59 ± 0.14) × 10^−10^	100–0	n/a	[324]
WPI: 40% milk fat fraction	(2.53 ± 0.50) × 10^−10^	100–0	n/a	[324]
WPI:40% BW	(1.25 ± 0.36) × 10^−10^	100–0	n/a	[324]
WPI:Gly:BW (60:20:20):PS 0.5 µm	(4.17 ± 0.69) × 10^−10^ ^a^	0	n/a	[325]
WPI:Gly:BW (30:10:60):PS 0.5 µm	(2.77 ± 1.39) × 10^−10^ ^a^	0	n/a	[325]
WPI:Gly:BW (30:10:60):PS 1.0 µm	(3.33 ± 0.56) × 10^−10^ ^a^	0	n/a	[325]
WPI:Gly:BW (30:10:60):PS 1.5 µm	(3.89 ± 0.56) × 10^−10^ ^a^	0	n/a	[325]
WPI:Gly:BW (30:10:60):PS 2.0 µm	(4.17 ± 0.83) × 10^−10^ ^a^	0	n/a	[325]
WPI control	(3.83 ± 4.72) × 10^−9^	100–50	n/a	[326]
WPI:Acetem	(5.55 ± 2.78)× 10^−11^	100–50	n/a	[326]
WPI control	(2.00 ± 0.03) × 10^−10^	100–0	(1.30 ± 0.28) × 10^−9^	[328]
WPI:Almond oil 0.5%	(1.56 ± 0.06) × 10^−10^	100–0	(1.55 ± 0.14) × 10^−9^	[328]
WPI:Almond oil 1.0%	(1.27 ± 0.19) × 10^−10^	100–0	(1.81 ± 0.27) × 10^−9^	[328]
WPI:Walnut oil 0.5%	(1.32 ± 0.19) × 10^−10^	100–0	(1.32 ± 0.16) × 10^−9^	[328]
WPI:Walnut oil 1.0%	(1.02 ± 0.09) × 10^−10^	100–0	(1.52 ± 0.66) × 10^−9^	[328]
WPI:Gly (1:1)	(3.32 ± 0.25) × 10^−11^	50–20	n/a	[329]
WPI:BW:Gly (1:1:1)	(2.58 ± 0.23) × 10^−11^	50–20	n/a	[329]
WPI:CW:Gly (1:1:1)	(2.62 ± 0.28) × 10^−11^	50–20	n/a	[329]
WPI control	(1.94 ± 0.28) × 10^−9^ ^a^	0	(3.18 ± 0.12) × 10^−9^ ^a^	[331]
WPI:Gly:CAN:7.5 g:100 g	(1.94 ± 0.28) × 10^−9^ ^a^	0	(3.30 ± 0.12) × 10^−9^ ^a^	[331]
WPI:Gly extruded	(1.25 ± 0.06) × 10^−8^ ^a^	0	n/a	[331]
WPI:Gly:CAN:7.5 g:100 g extruded	(9.72 ± 0.28) × 10^−9^ ^a^	0	n/a	[331]
WPI:Gly compressed extruded	(3.61 ± 0.28) × 10^−9^ ^a^	0	(2.89 ± 0.58) × 10^−10^ ^a^	[331]
WPI:Gly:CAN:7.5 g:100 g compressed extruded	(2.28 ± 0.56) × 10^−9^ ^a^	0	(3.47 ± 0.58) × 10^−10^ ^a^	[331]
WPI:TiO_2_ 0% *w*/*w*	(3.19 ± 0.08) × 10^−10^	n/a	n/a	[281]
WPI:TiO_2_ 1% *w*/*w*	(2.89 ± 0.11) × 10^−10^	n/a	n/a	[281]
WPI:TiO_2_ 0 wt %	(2.78 ± 0.28) × 10^−9^ ^a^	100–50	n/a	[282]
WPI:TiO_2_ 1 wt %	(2.42 ± 0.28) × 10^−9^ ^a^	100–50	n/a	[282]
WPI:TiO_2_ 4 wt %	(1.02 ± 0.83) × 10^−9^ ^a^	100–50	n/a	[282]
WPI control	(66.0 ± 3.6) × 10^−9^	68.8 ± 1.3	n/a	[284]
WPI:Cloisite Na^+^	(47.1 ± 3.3) × 10^−9^	72.2 ± 0.9	n/a	[284]
WPI:Cloisite 30B	(55.6 ± 7.5) × 10^−9^	70.1 ± 0.8	n/a	[284]
WPI:Cloisite 20A	(64.8 ± 4.2) × 10^−9^	71.1 ± 1.4	n/a	[284]
WPI:ZNP 0 *w*/*w*	(8.94 ± 0.25) × 10^−11^	25 ± 1	n/a	[285]
WPI:ZNP 0.4 *w*/*w*	(3.42 ± 0.33) × 10^−11^	26 ± 1	n/a	[285]
WPI:ZNP 1.2 *w*/*w*	(1.44 ± 0.56) × 10^−12^	27 ± 1	n/a	[285]
WPI control	n/a	50 ± 1	(2.53 ± 0.16) × 10^−9^	[308]
WPI:lactoperoxidase system 0.7% *w*/*w*	n/a	50 ± 1	(2.56 ± 0.19) × 10^−9^	[308]
WPI:Sorbitol (50:50)	2.68 × 10^−9^	50–25	n/a	[315]
WPI:Sorbitol:BW (50:35:15)	1.49 × 10^−9^	50–25	n/a	[315]
WPI:Sorbitol:Potassium sorbate (50:43.3:6.7)	3.03 × 10^−9^	50–25	n/a	[315]
WPI:Sorbitol:BW:Potassium sorbate (50:35:11.7:3.3)	2.34 × 10^−9^	50–25	n/a	[315]
SPI:WG (2:1):pH 7.0	0.50 × 10^−9^ ^a^	55	2.43 × 10^−11^	[345]
SPI:WG (2:1):cys:pH 7.0	0.90 × 10^−9^ ^a^	55	2.55 × 10^−11^	[345]
SPI:WG (3:1):pH 7.0	0.85 × 10^−9^ ^a^	55	0.61 × 10^−11^	[345]
SPI:WG (3:1) cys:pH 7.0	0.75 × 10^−9^ ^a^	55	1.13 × 10^−11^	[345]
SPI:WG (4:1):pH 7.0	0.50 × 10^−9^ ^a^	55	1.57 × 10^−11^	[345]
SPI:WG (4:1) cys:pH 7.0	0.55 × 10^−9^ ^a^	55	1.39 × 10^−11^	[345]
SPI:WG (1:0):pH 7.0	2.7 × 10^−9^ ^a^	55	0.67 × 10^−11^	[345]
SPI:WG (1:0) cys:pH 7.0	2.5 × 10^−9^ ^a^	55	0.57 × 10^−11^	[345]
SPI:DAS 0%	(15.0 ± 0.2) × 10^−10^	73.1 ± 0.3	n/a	[340]
SPI:DAS 10%	(16.5 ± 0.89) × 10^−10^	72.7 ± 0.5	n/a	[340]
SPI:10% fatty acid	(28 ± 0.94) × 10^−10^	100–50	n/a	[343]
SPI:10% lauric acid	(17.5 ± 1.11) × 10^−10^	100–50	n/a	[343]
SPI:10% myristic acid	(20.6 ± 0.42) × 10^−10^	100–50	n/a	[343]
SPI:10% palmitic acid	(22.4 ± 1.44) × 10^−10^	100–50	n/a	[343]
SPI:10% oleic acid	(18.5 ± 0.69) × 10^−10^	100–50	n/a	[343]
SPI:BW:Span	2.22 × 10^−10^	54	0	[341]
WG:Gly (83.3:16.7)	(9.5 ± 0.9) × 10^−11^	100–0	n/a	[3]
WG:Gly:BW (66.7:16.7:20)	(3.5 ± 0.17) × 10^−11^	100–0	n/a	[3]
WG:Gly:carnauba wax (66.7:16.7:20)	(6.51 ± 0.43) × 10^−11^	100–0	n/a	[3]
WG:Gly:refined paraffin (66.7:16.7:20)	(6.9 ± 0.43) × 10^−11^	100–0	n/a	[3]
WG:Gly:oleic acid (66.7:16.7:20)	(7.8 ± 0.439) × 10^−11^	100–0	n/a	[3]
WG:Gly:soy lecithin (66.7:16.7:20)	(1.0 ± 0.1) × 10^−10^	100–0	n/a	[3]
WG:Gly:acetic ester of monoglyceride (66.7:16.7:20)	(1.2 ± 0.1) × 10^−10^	100–0	n/a	[3]
WG:Gly:diacetyl tartaric ester of monoglyceride (66.7:16.7:20)	(4.3 ± 0.1) × 10^−11^	100–0	n/a	[3]
WG:Gly:sucroglyceride (66.7:16.7:20)	(5.64 ± 0.17) × 10^−11^	100–0	n/a	[3]
WG:Gly:stearic alcohol (66.7:16.7:20)	(4.34 ± 0.17) × 10^−11^	100	n/a	[3]
WG:MMT 5.0 wt %	6.5 × 10^−12^ ^a^	100	8.0 × 10^−12^ ^a^	[300]
WG:MMT 7.5 wt %	7.0 × 10^−12^ ^a^	100	8.1 × 10^−12^ ^a^	[300]
WG:MMT 10.0 wt %	6.0 × 10^−12^ ^a^	100	8.3 × 10^−12^ ^a^	[300]
WG control	(3.0 ± 0.1) × 10^11^	n/a	n/a	[301]
WG:MMT 2.5% C_0_	(2.2 ± 0.1) × 10^11^	n/a	n/a	[301]
WG:MMT 5.0% C_0_	(1.8 ± 0.1) × 10^11^	n/a	n/a	[301]
**Nanotechnology**				
SPI control	(4.3 ± 0.2) × 10^−10^	65	n/a	[290]
SPI:SNC 5%	(4.8 ± 0.3) × 10^−10^	65	n/a	[290]
SPI:SNC 20%	(3.9 ± 0.1) × 10^−10^	65	n/a	[290]
SPI:SNC 40%	(3.57 ± 0.08) × 10^−10^	65	n/a	[290]
SPI:Cloisite 20A 0%	(10.56 ± 0.31) × 10^−10^	100–65	n/a	[295]
SPI:Cloisite 20A 5%	(7.31 ± 0.08) × 10^−10^	100–65	n/a	[295]
SPI:Cloisite 20A 10%	(6.00 ± 0.28) × 10^−10^	100–65	n/a	[295]
SPI:Cloisite 20A 15%	(5.69 ± 0.31) × 10^−10^	100–65	n/a	[295]
SPI:Cloisite 30B 0%	(10.56 ± 0.31) × 10^−10^	100–65	n/a	[295]
SPI:Cloisite 30B 5%	(8.58 ± 0.14) × 10^−10^	100–65	n/a	[295]
SPI:Cloisite 30B 10%	(7.42 ± 0.22) × 10^−10^	100–65	n/a	[295]
SPI:Cloisite 30B 15%	(6.47 ± 0.25) × 10^−10^	100–65	n/a	[295]
SPI control	(10.56 ± 0.31) × 10^−10^	100–65	n/a	[295]
SPI:MMT 5%	(8.22 ± 0.28) × 10^−10^	100–65	n/a	[295]
SPI:MMT 10%	(6.92 ± 0.22) x 10^−10^	100–65	n/a	[295]
SPI:MMT 15%	(6.03 ± 0.17) x 10^−10^	100–65	n/a	[295]
SPI:MMT 0%	(12 ± 0.8) × 10^−11^	75	n/a	[298]
SPI:MMT 2.5%	(11 ± 0.3) × 10^−11^	75	n/a	[298]
SPI:MMT 5%	(6.8 ± 0.2) × 10^−11^	75	n/a	[298]
SPI:MMT 7.5%	(5.0 ± 0.6) × 10^−11^	75	n/a	[298]
SPI:MMT 10%	(3.2 ± 0.9) × 10^−11^	75	n/a	[298]
SPI control	(1.21 ± 0.04) × 10^−6^	100	n/a	[299]
SPI:PNP 0.5%	(1.06 ± 0.04) × 10^−6^	100	n/a	[299]
SPI:PNP 1.0%	(1.00 ± 0.03) × 10^−6^	100	n/a	[299]
SPI:PNP 2.0%	(0.92 ± 0.02) × 10^−6^	100	n/a	[299]
SPI:PNP 4.0%	(0.83 ± 0.01) × 10^−6^	100	n/a	[299]
**Antimicrobial Films**				
SPI control	(14.9 ± 0.5) × 10^−11^	65	n/a	[318]
SPI:PLA (60:40)	(3.4 ± 0.1) × 10^−11^	65	n/a	[318]
SPI:PLA (50:50)	(2.3 ± 0.1) × 10^−11^	65	n/a	[318]
SPI control	(0.47 ± 0.03) × 10^−3^	54	n/a	[322]
SPI:Rutin	(0.33 ± 0.06) × 10^−3^	54	n/a	[322]
SPI:Epicatechin	(0.64 ± 0.06) × 10^−3^	54	n/a	[322]

Some data are converted to the same units. ^a^ Numerical value estimated from graph; ^b^ Water Vapour Transmission Rate in g·m^−2^·d^−1^; n/a not available.
